# Autism-related proteins form a complex to maintain the striatal asymmetry in mice

**DOI:** 10.1038/s41422-025-01174-9

**Published:** 2025-09-02

**Authors:** Yisheng Jiang, Feipeng Zhu, Jie Zhong, Xiaomei Sun, Yuting Yuan, Shuo Wang, Haiyang Chen, Zhiheng Xu

**Affiliations:** 1 Second Affiliated Hospital, Zhejiang University School of Medicine, Hangzhou, Zhejiang China; 2 Institute of Genetics and Developmental Biology, Chinese Academy of Sciences, Beijing, China; 3 University of Chinese Academy of Sciences, Beijing, China

**Keywords:** Phosphorylation, Phosphorylation, Mechanisms of disease, Cell signalling

## Abstract

The brain’s hemispheres exhibit profound lateralization, yet the underlying mechanisms remain elusive. Using proteomic and phosphoproteomic analyses of the bilateral striatum — a hub for important brain functions and a common node of autism pathophysiology — we identified significant phosphorylation asymmetries. Particularly, the phosphorylation processes in the left striatum appear more prone to disturbance. Notably, SH3RF2, whose single-copy knockout leads to autism spectrum disorder (ASD)-like behaviors in mice, is uniquely expressed in the striatum, forming a complex with CaMKII (an ASD-associated protein) and PPP1CC. Loss of SH3RF2 disturbs the CaMKII/PP1 “switch”, resulting in hyperactivity of CaMKII and increased phosphorylation of its substrate GluR1. In *Sh3rf2*-deficient mice, heightened GluR1-Ser831 phosphorylation and its aberrant postsynaptic membrane localization in the left striatum may impair the functional lateralization of striatal neurons and contribute to autism-like behaviors. This study unveils the first molecular mechanism governing brain lateralization in mammals, linking its impairment to autism development and treatment strategies.

## Introduction

The structural and functional differences between the left and right hemispheres are fundamental aspects of brain organization and are evolutionarily conserved.^1^ In mammals, particularly humans, brain lateralization plays a vital role in advanced neurological functions, such as language, speech, memory, consciousness and cognition.^1^ For example, it was discovered more than 160 years ago that damage to Broca’s area in the left hemisphere leads to expressive aphasia.^2^ Later, lesions in the right hemisphere were found to result in diminished emotional expression and inappropriate apathy.^3^ Abnormalities in brain lateralization are associated with various neuropsychiatric disorders, including autism spectrum disorder (ASD), schizophrenia, and Parkinson’s disease.^4–6^ Although scientists have endeavored to uncover the mysteries of brain lateralization, only recent studies have revealed some mechanisms in zebrafish epithalamus asymmetry.^7–10^ In more complex mammalian species, the molecular bases of brain lateralization and the causal nexus between its abnormalities and disorders like ASD remain enigmatic. Answering the above two fascinating and long-standing questions will be of great relevance.

ASD, characterized by impaired social interactions and repetitive stereotyped behaviors, is one of the most prevalent mental disorders in children.^11^ Emerging evidence suggests that atypical brain lateralization in ASD patients may represent a neurobiological underpinning of core symptoms. Neuroimaging studies have revealed widespread abnormalities in gray and white matter lateralization, including reduced asymmetry in higher-order association cortices and language-related regions.^12,13^ At the functional level, ASD patients exhibit significantly reduced left-lateralization in language-related brain regions (e.g., Broca’s area and superior temporal gyrus), often displaying bilateral or even right-lateralized activation, with the greater reduction correlating with symptom severity.^14^ Additionally, individuals with ASD often exhibit abnormal functional activity of social cognitive networks alongside reduced connectivity within default mode network in the right hemisphere.^15,16^ Notably, altered lateralization patterns within the striatum have been reported in ASD patients.^12,17,18^ The human caudate nucleus, analogous to the dorsomedial striatum (DMS) in rodents,^19^ exhibits inherent rightward volumetric lateralization.^20^ This characteristic neural asymmetry becomes markedly attenuated in ASD populations,^12^ implicating striatal dysfunction in pathogenesis. However, current studies remain largely phenomenological, with mechanistic insights awaiting elucidation.

Our previous study demonstrated that haploinsufficiency of *Sh3rf2* causes unilateral hippocampal disturbances and ASD-like behaviors in mice.^21^ SH3RF2 was indicated as a regulator of serine/threonine phosphatase-1 (PP1).^22^ PP1 and Ca^2+^/calmodulin-dependent protein kinase II (CaMKII) form a synaptic “switch” (CaMKII/PP1) to regulate Ca^2+^-mediated neuronal activities.^23,24^ PP1’s activity and functional specificity depend on regulatory subunits that control substrate access.^25^ Although the CaMKII/PP1 “switch” is well-known, the regulator of CaMKII dephosphorylation in the postsynaptic density (PSD) remains unknown, hindering our understanding of synaptic plasticity. Moreover, SH3RF2’s molecular role and its link to ASD-like behaviors are unresolved.

In this study, we reveal pronounced interhemispheric disparities in striatal phosphoproteomic architecture, including multiple autism-associated proteins. DMS DRD1 neurons exhibit intrinsic structural and functional rightward lateralization, a phenomenon eliminated in *Sh3rf2* knockout (KO) mice. Remarkably, chemogenetic suppression of activity in DRD1 neurons in the left DMS partially rescues ASD-like behaviors of KO mice. Mechanistically, SH3RF2 orchestrates CaMKII/PP1 complex assembly to modulate CaMKII activity, with its loss disrupting unilateral phosphorylation control, consequently impairing bilateral neural specialization and inducing ASD-like behaviors.

## Results

### Asymmetry of protein phosphorylation across the bilateral striatum

**Fig. 1:**
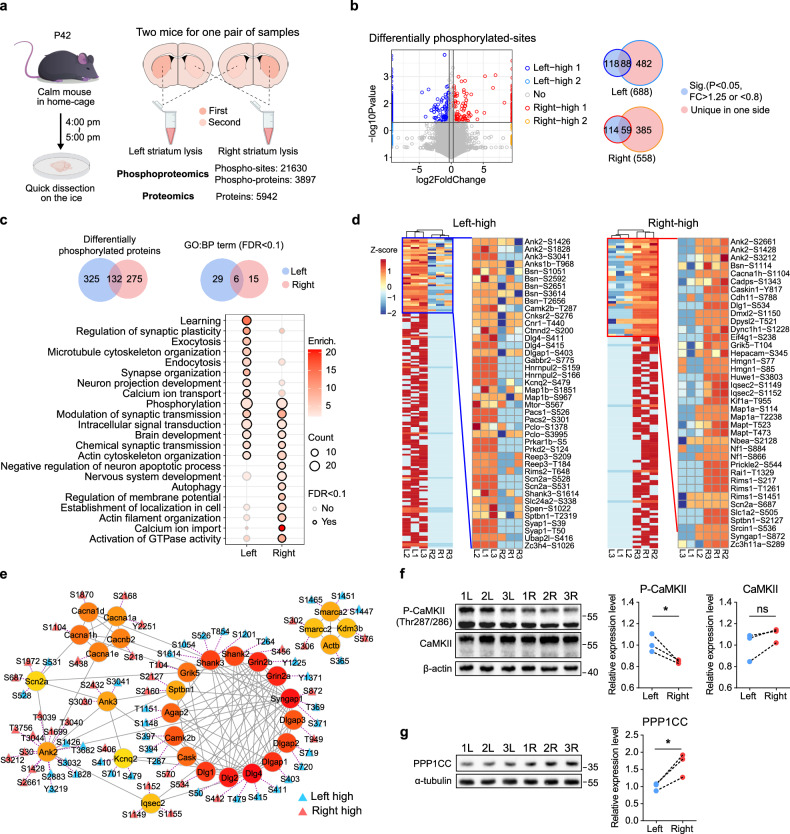
Asymmetries in protein phosphorylation in bilateral striatum reveled by proteomics and phosphoproteomics. **a** P42 WT C57BL/6J mice were utilized for the omics studies. Each pair of striatum specimen was derived from two mice. **b** Volcano plot and Venn diagram displayed the number of phosphorylation sites with significantly differential levels between the left and right striatum. These sites exhibit statistical significance (*P* value < 0.05, fold change > 1.25 or < 0.8) between two sides or are detected exclusively on one side (in at least two samples from the same side). **c** Number of differentially phosphorylated proteins in the left and right striatum and their corresponding GO terms. **d** Heatmaps illustrated the phosphorylation sites of autism-related proteins with differential phosphorylation levels in the left and right striatum. **e** Hub analysis of PPI network of autism-related proteins with differentially phosphorylated sites in the left and right striatum. The color filled within the circles represents the rank of the proteins in the hub analysis. The redder the color, the more central the protein is within the network. **f**, **g** Western blot and quantitative results displayed the expression levels of total CaMKII and phosphorylated CaMKII (**f**) and PPP1CC (**g**) in the bilateral striatum. The specimens were the same as those in **a**. Paired *t*-test. All data are presented as mean ± SEM; *P < 0.05; ns no significance.

Given the striatum’s pivotal role in orchestrating multifaceted neural functions and its strong association with ASD, we conducted an investigation into bilateral striatal disparities using integrated proteomic and recently improved phosphoproteomic analyses.^26,27^ A total of 5942 proteins and 21,630 phosphorylation sites across 3897 phosphoproteins were identified in the dorsal striatum of wild-type (WT) mice (Fig. 1a). Considering the subtle differences between the two sides of striatum, proteins and phosphorylation sites either detected exclusively in one side (in at least two samples) or showing fold change > 1.25 or < 0.8 and *P*-value < 0.05 were deemed significantly different. We identified 688 phosphorylation sites with higher phosphorylation level in the left striatum and 558 in the right striatum (Fig. 1b; Supplementary information, Table S1). Gene ontology (GO) analysis of the 325 left-higher and 275 right-higher phosphorylated proteins revealed that both sets of proteins are involved in phosphorylation, modulation of synaptic transmission, intracellular signal transduction, brain development, chemical synaptic transmission and actin cytoskeleton organization processes, underscoring the neural activity differences in the bilateral striatum (Fig. 1c; Supplementary information, Table S2). Notably, each side of striatum has some biological processes with lateralization, such as learning on the left side and activation of GTPase activity on the right. In addition, we found 42 proteins with higher expression in the left striatum and 38 in the right striatum (Supplementary information, Fig. S1a and Table S3). Gene set enrichment analysis (GSEA) of the proteome data indicated that the left-higher proteins are involved in anion channel activity, RNA 3′-end processing and nuclear chromosome telomeric region (Supplementary information, Fig. S1b), whereas the right-higher proteins primarily participate in the regulation of protein phosphorylation (Supplementary information, Fig. S1c). Furthermore, the left striatum exhibited more phosphorylated serine sites and peptides with dual phosphorylation (Supplementary information, Fig. S1d, e). These results suggested that the basal phosphorylation levels in the left striatum are higher.

### Asymmetric phosphorylation of ASD-associated proteins between the bilateral striatum

To investigate the relationship between ASD and hemispheric asymmetry, we focused on autism-related proteins. We identified 178 phosphorylation sites with higher phosphorylation level on the left striatum and 124 on the right among 142 autism-related proteins, including ANK2, CaMK2B and SHANK3 (Fig. 1d; Supplementary information, Fig. S1f, g). Importantly, *χ*^2^ analysis confirmed that these differentially phosphorylated proteins were significantly enriched for autism-related genes (Supplementary information, Fig. S1h). Additionally, we found 5 autism-related proteins with higher expression on the left and 5 on the right (Supplementary information, Fig. S1i, j). We then queried the STRING database for protein–protein interactions (PPIs) of these asymmetrically phosphorylated autism-related proteins and performed a hub analysis.^28^ Notably, the asymmetric phosphorylation of most postsynaptic proteins, such as SHANK2, SHANK3 and CaMK2B, was left-higher (Fig. 1e). CaMKII is a pivotal kinase in the PSD, and Thr287 of CaMKII β-isoform (corresponding to Thr286 in α-isoform) is an autophosphorylation site crucial for maintaining enzyme activity in the absence of elevated Ca^2+^ and inducing long-term potentiation in neurons.^29,30^ We confirmed that the phosphorylation level of CaMK2B-Thr287 was higher in the left striatum (Fig. 1f), whereas the PP1 catalytic subunit gamma (PPP1CC) level was higher in the right striatum (Fig. 1g). Given that PPP1CC is specifically concentrated in the PSD and negatively regulates the phosphorylation of CaMK2B-Thr287,^31^ this result suggested that phosphorylation process in the right striatum is under more stringent regulation.

### SH3RF2 is involved in postsynaptic signaling of striatal MSNs

**Fig. 2:**
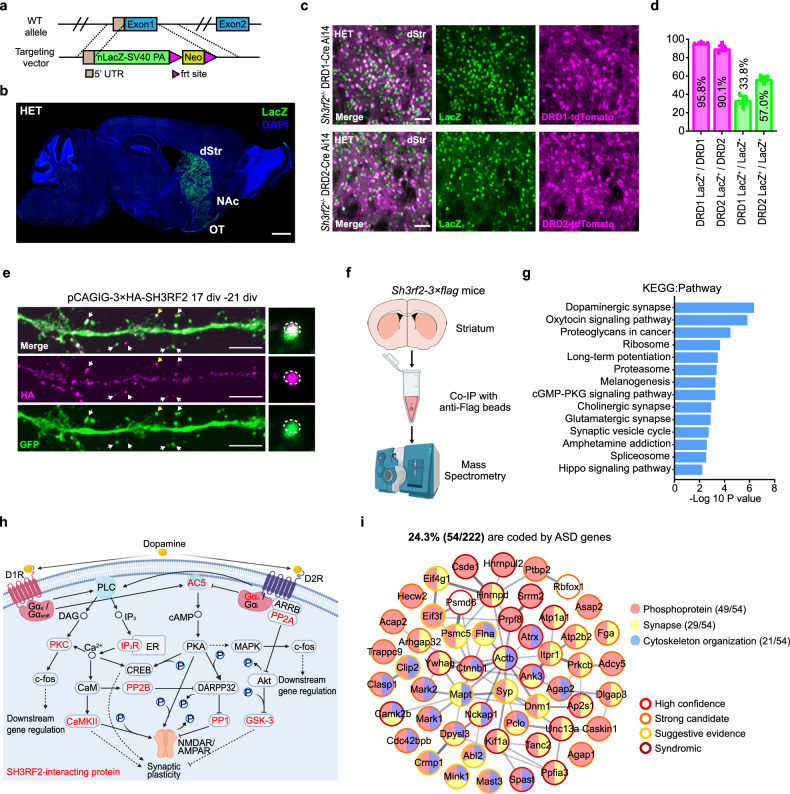
Expression profile of SH3RF2 and its potential functions. **a** Schematic diagram of *Sh3rf2* KO mice. nLacZ-SV40-polyA (PA) was inserted to replace the first exon of *Sh3rf2*. **b** Sagittal brain slice of *Sh3rf2*^*+/–*^ mice was immunostained with anti-LacZ antibody and DAPI. dStr: dorsal striatum; NAc: nucleus accumbens; OT: olfactory tubercle. Scale bar, 1000 μm. **c** Coronal striatum sections of *Sh3rf2*^*+/–*^ mice with DRD1-MSNs (top panel) or DRD2-MSNs (bottom panel) labeled by tdTomato were immunostained with anti-LacZ antibody. Scale bars, 50 μm. **d** Quantitative analysis of co-localization of LacZ with DRD1-tdTomato and DRD2-tdTomato in the dorsal striatum. *n* = 3 slices. **e** Cultured striatal neuron expressing 3× HA-SH3RF2 (transfected at 17 days in vitro (div) and fixed at 21 div) were immunostained with anti-HA and anti-GFP antibodies. Arrows: spines containing SH3RF2. The spines marked by yellow arrows were magnified. Scale bars, 5 μm. **f** Detect SH3RF2-interacting proteins using mass spectrometry. **g** KEGG pathway analysis of SH3RF2-interacting proteins. The dopaminergic synapse is the top 1 term. **h** Dopaminergic synapse pathway (referred to KEGG mmu04728 and created in BioRender.com). Red font: SH3RF2-interacting proteins detected in mass spectrum analysis. **i** PPI network of SH3RF2-interacting proteins associated with ASD. The confidences of ASD-risk proteins were referred to SFARI (https://gene.sfari.org/). PPI network and GO analysis were performed on STRING (https://cn.string-db.org/). The thickness of the lines between proteins indicates the confidence index of the interaction.

Since SH3RF2 regulates the PP1 activity and may be pertinent to lateralized brain function, we proceeded to inspect its spatiotemporal expression patterns in the mouse brain. In *Sh3rf2*^*+/–*^ (heterozygous, HET) mice, anti-LacZ antibody was used to detect *Sh3rf2* expression, as its first exon is replaced by the β-galactosidase gene (Fig. 2a). Immunofluorescence and western blot results revealed that *Sh3rf2* is predominantly expressed in the striatum, including the dorsal part (dStr), nucleus accumbens (NAc) and olfactory tubercle (OT) (Fig. 2b; Supplementary information, Fig. S2a–d). We found that all LacZ^+^ cells were NeuN^+^ (a marker of neuron), and 93.6% of striatal neurons expressed LacZ, indicating that *Sh3rf2* is specifically expressed in striatal neurons (Supplementary information, Fig. S2e, f). Within the striatum, > 90% of striatal neurons are GABAergic medium spiny neurons (MSNs) expressing dopamine receptor type 1 (DRD1) or 2 (DRD2).^32^ We crossed *Sh3rf2*^*+/–*^ mice with Drd1a-Cre/Ai14 or Drd2-Cre/Ai14 strains to fluorescently label MSN subtypes, and found that 95.8% of DRD1-MSNs and 90.1% of DRD2-MSNs were LacZ^+^, and 90.8% of LacZ^+^ cells comprised DRD1-MSNs (33.8%) and DRD2-MSNs (57.0%) (Fig. 2c, d). Meanwhile, *Sh3rf2* is not expressed in cholinergic interneurons (Chat^+^) or GABAergic interneurons (PV^+^ or SOM^+^) (Supplementary information, Fig. S2g). To determine the subcellular localization of SH3RF2, we overexpressed it in cultured MSNs and found that SH3RF2 is localized in dendrites and concentrated in the PSD, exhibiting a pattern analogous to that of CaMKII and PPP1CC (Fig. 2e; Supplementary information, Fig. S2h).

To investigate the molecular function of SH3RF2, we generated *Sh3rf2-3*× *flag* knock-in mice via CRISPR/Cas9 and verified that SH3RF2 protein is predominantly expressed in the striatum (Supplementary information, Fig. S3a, b). We observed that the expression level of SH3RF2 in the striatum initially ascended and subsequently declined with the age of the mice, peaking at postnatal days P24–30 (Supplementary information, Fig. S3c, d). In humans, SH3RF2 is also expressed primarily in the striatum, with its temporal expression pattern recapitulating the murine pattern, suggesting evolutionary conservation of SH3RF2 function (Supplementary information, Fig. S3e). Then, we identified potential SH3RF2-interacting proteins in the striatum of *Sh3rf2-3*× *flag* knock-in mice through co-immunoprecipitation (co-IP) and mass spectrometry (Fig. 2f; Supplementary information, Table S4). PPI network combined with GO analysis revealed that a cohort of SH3RF2-interacting proteins participate in protein phosphorylation and dephosphorylation, predominantly targeting serine/threonine sites (Supplementary information, Fig. S4a, b). KEGG pathway analysis revealed SH3RF2 association with multiple kinases, including CaMK2B and PKC, as well as phosphatases such as PPP1CC and PPP2CB (PP2A), in the dopaminergic synapse pathway (Fig. 2g, h). Remarkably, 24.3% (54/222) of the SH3RF2-interacting proteins are encoded by ASD risk genes, a significant enrichment confirmed by *χ*^2^ analysis, with the majority (49/54) possessing at least one phosphorylation site (Fig. 2i; Supplementary information, Fig. S4c, d).

### *Sh3rf2* deletion disrupts asymmetric phosphorylation of CaMKII between the bilateral striatum

**Fig. 3:**
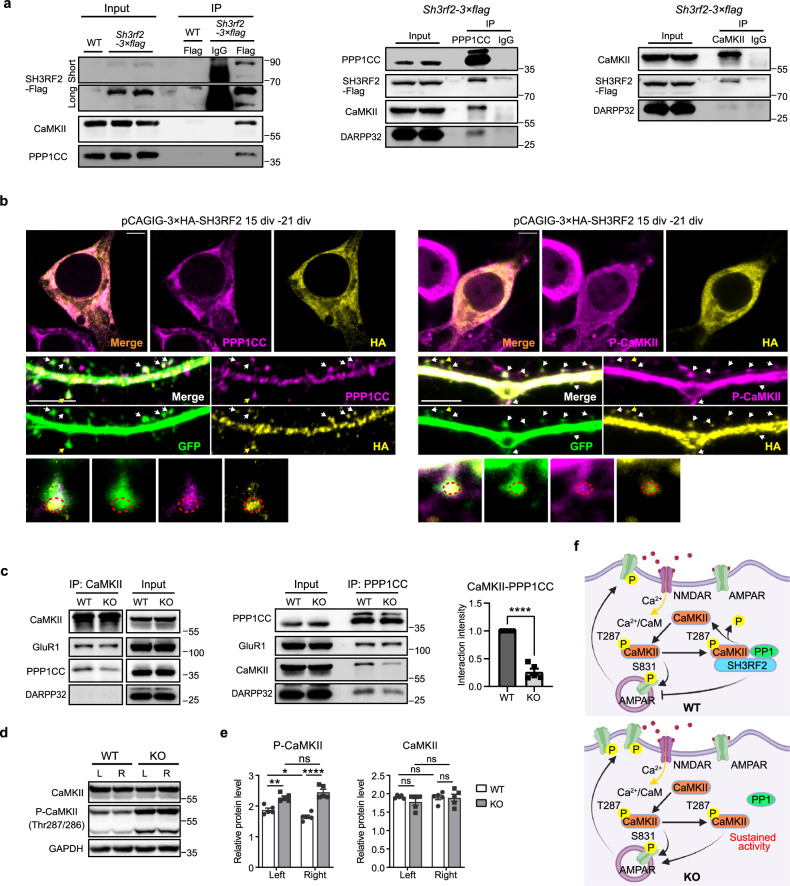
The SH3RF2/CaMKII/PPP1CC protein complex. **a** Co-IP assay indicated that SH3RF2 can interact with CaMKII and PPP1CC. Short: short exposure; Long: long exposure. **b** Immunostaining of cultured striatal neurons expressing 3× HA-SH3RF2 (transfected at 15 div, fixed at 21 div) using antibodies against HA, PPP1CC, and Thr287/286-phosphorylated CaMKII (p-CaMKII). Arrows: spines with co-localization of SH3RF2 with PPP1CC (left panel) and with p-CaMKII (right panel). Spines marked with yellow arrows were magnified. Scale bars, 5 μm. **c** Co-IP assay and quantitative result showed reduced interaction between CaMKII and PPP1CC in the striatum of *Sh3rf2* KO mice. *n* = 4 mice per group. One-sample *t*-tests compared normalized mean of KO against the theoretical value of 1 (representing WT). **d**, **e** Western blot and quantitative results displayed the expression levels of total CaMKII and p-CaMKII in the left and right striatum. *n* = 5 mice per group. Linear mixed model (genotype × hemisphere + (1|batch)) with ANOVA and Tukey post-hoc tests. **f** Model of the role of SH3RF2/CaMKII/PPP1CC protein complex in synapses. All data are presented as mean ± SEM; *P < 0.05; **P < 0.01; ****P < 0.0001; ns no significance.

Our findings demonstrated an asymmetrical distribution of CaMK2B-Thr287 phosphorylation and PPP1CC expression between the bilateral striatum. Consequently, we investigated whether SH3RF2 contributes to striatal lateralization by regulating this CaMKII/PP1 “switch”. Endogenous co-IP confirmed that SH3RF2 can interact with CaMKII and PPP1CC in the striatum (Fig. 3a). Consistent with these interactions, immunostaining in cultured striatal neurons showed that SH3RF2 was highly colocalized with PPP1CC and Thr287/286-phosphorylated CaMKII in the soma and dendritic spines (Fig. 3b). These findings suggested a role for SH3RF2 in promoting PP1-dependent dephosphorylation of CaMKII. To determine whether SH3RF2 acts as a scaffold protein mediating the dephosphorylation of CaMKII by PPP1CC in the PSD, we examined CaMKII–PPP1CC interaction with co-IP. Striatal tissue from *Sh3rf2* KO mice exhibited a marked reduction (~80%) in CaMKII–PPP1CC complex formation compared to WT mice (Fig. 3c; Supplementary information, Fig. S4e). Notably, the phosphorylation levels of CaMKII-Thr287/286 became comparable in the bilateral striatum of KO mice, thereby abolishing the intrinsic asymmetric phosphorylation pattern characteristic of bilateral striatum observed in WT mice (Fig. 3d, e).

Collectively, these findings implied that the SH3RF2/CaMKII/PPP1CC complex orchestrates asymmetric phosphorylation in diverse postsynaptic proteins within the striatum, encompassing those implicated in ASD. We proposed a model in which loss of SH3RF2 causes CaMKII to maintain phosphorylation at Thr287/286 and kinase activity even in the absence of elevated Ca^2+^ level, potentially inducing the phosphorylation of α-amino-3-hydroxy-5-methylisoxazole-4-propionic acid receptor (AMPAR) subunit, GluR1, which is a direct target of CaMKII.^33,34^ Since phosphorylation of GluR1-Ser831 can promote AMPAR targeting to PSD and recruitment to the postsynaptic membrane,^35–37^ loss of SH3RF2 may affect AMPAR function (Fig. 3f).

### *Sh3rf2* deletion abolishes the asymmetric expression of GluR1 in the PSD between the bilateral striatum

**Fig. 4:**
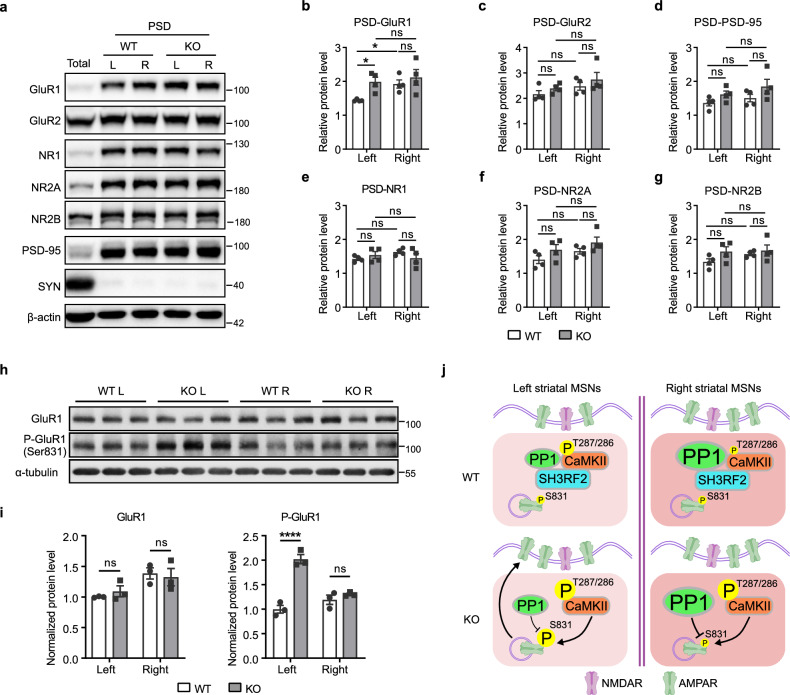
SH3RF2/CaMKII/PPP1CC complex regulates the asymmetric expression of GluR1 on PSD. **a** Representative western blot results demonstrated the expression levels of AMPAR and NMDAR subunits in the PSD fraction of left and right striatum. SYN (synaptophysin) serves as a negative control. **b**–**g** Quantitative results displayed the expression levels of AMPAR and NMDAR subunits in the PSD fraction. *n* = 4 mice per group. Linear mixed model (genotype × hemisphere + (1|batch)) with ANOVA and Tukey post-hoc tests. **h**, **i** Western blot and quantitative results displayed the protein levels of total GluR1 and phosphorylated GluR1 in the left and right striatum. *n* = 3 mice per group. Two-way ANOVA with Sidak’s multiple comparisons test. **j** Molecular mechanism by which SH3RF2 deficiency leads to impaired functional lateralization of striatal MSNs. All data are presented as mean ± SEM; *P < 0.05; ****P < 0.0001; ns no significance.

To investigate whether AMPARs are affected by *Sh3rf2* deletion, we performed subcellular fractionation of striatal tissue. Our findings revealed that the expression level of PSD-localized GluR1 (PSD-GluR1) was higher in the right vs left striatum of WT mice (Fig. 4a, b). Notably, in *Sh3rf2* KO mice, the expression of PSD-GluR1 in the left striatum was significantly elevated, reaching levels comparable to the right striatum and thereby disrupting the asymmetric expression of PSD-GluR1 observed in WT mice (Fig. 4a, b). The expression levels of other postsynaptic proteins — including PSD-95, GluR2, and the *N*-methyl-D-aspartate receptor (NMDAR) subunits NR1, NR2A, and NR2B — showed no significant differences between the left and right striatum and were unaffected by *Sh3rf2* deletion (Fig. 4a, c–g). Intriguingly, GluR1-Ser831 phosphorylation was specifically elevated in the left striatum of *Sh3rf2* KO mice relative to WT controls, while total GluR1 level remained unaltered (Fig. 4h, i).

Collectively, we proposed that SH3RF2 is involved in the molecular mechanism of striatal lateralization by forming a complex with CaMKII and PPP1CC (Fig. 4j). Compared to the left striatum, PPP1CC expression is higher and the basal phosphorylation level of CaMKII-Thr287/286 is lower in the right striatum. Additionally, MSNs in the right striatum have more postsynaptic AMPARs. When SH3RF2 is absent, CaMKII loses the regulation by PPP1CC, resulting in an elevated phosphorylation level of CaMKII-Thr287/286. Activated CaMKII further phosphorylates Ser831 of the AMPAR subunit GluR1. However, due to the higher expression of PPP1CC in the right striatum, the phosphorylation process is more stringently regulated, potentially causing the increase in GluR1-Ser831 phosphorylation to occur only in the left striatum.^38,39^ This promotes the recruitment of AMPARs to the postsynaptic membrane, thereby disrupting the original asymmetry of AMPARs and consequently impairing striatal functional lateralization.

### *Sh3rf2* deletion disrupts the rightward lateralization of striatal DRD1-MSNs

**Fig. 5:**
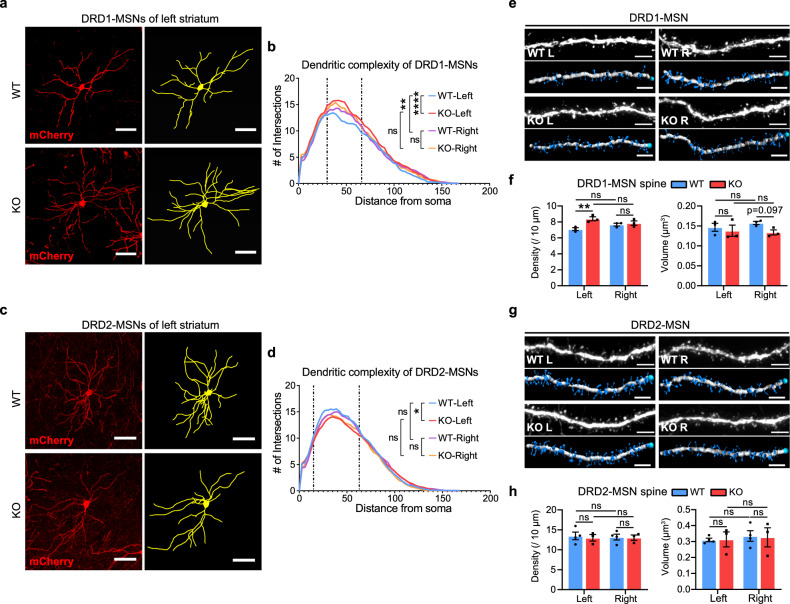
SH3RF2 deficiency induces divergent alterations in the dendritic complexity and spine morphology of DRD1-MSNs and DRD2-MSNs in the left and right striatum. **a**, **c** Representative fluorescent images (left) and reconstructed depictions (right) of DRD1-MSNs (**a**) and DRD2-MSNs (**c**) sparsely labeled by AAV in the left striatum of WT and *Sh3rf2* KO mice. Scale bars, 50 μm. **b**, **d** Sholl analysis of dendritic complexity of DRD1-MSNs (**b**) and DRD2-MSNs (**d**). Statistical analysis of the curves within the range of 30–66 μm (**b**) or 15–63 μm (**d**). *n* = 3 or 4 mice for each group, and 5–8 neurons for each mouse. One-way ANOVA with Tukey post-hoc tests. **e**, **g** Representative fluorescent images and reconstructed depictions of DRD1-MSN spines (**e**) and DRD2-MSN spines (**g**). Scale bars, 5 μm. **f**, **h** Quantitative analysis of the density (left) and volume (right) of DRD1-MSN spines (**f**) and DRD2-MSN spines (**h**). *n* = 3 or 4 mice for each group. Each dot represents a mouse. Linear mixed model (genotype × hemisphere + (1|mouse)) with ANOVA and Tukey post-hoc tests. All data are presented as mean ± SEM; *P < 0.05; **P < 0.01; ****P < 0.0001; ns no significance.

We next examined whether SH3RF2 plays a role in striatal lateralization. Utilizing adeno-associated virus (AAV), we sparsely labeled striatal DRD1-MSNs and DRD2-MSNs, respectively, in the DMS (Supplementary information, Fig. S5a, b). In *Sh3rf2* KO mice, the dendritic complexity of DRD1-MSNs in the left striatum was significantly increased, whereas no such increase was observed in the right striatum (Fig. 5a). This change abolished the significantly greater dendritic complexity of DRD1-MSNs in the right vs left striatum, a characteristic feature of WT mice (Fig. 5b). In contrast, the dendritic complexity of DRD2-MSNs in *Sh3rf2* KO mice was significantly decreased in the left striatum but remained unchanged in the right striatum, with no notable left-right differences in either WT or KO mice (Fig. 5c, d).

Subsequently, we reconstructed the dendritic spines of DRD1-MSNs and DRD2-MSNs and assessed their density and volume. In *Sh3rf2* KO mice, DRD1-MSN spine density was significantly increased in the left striatum, while spine volume exhibited a decreasing trend in the right striatum (Fig. 5e, f; Supplementary information, Fig. S5c). An increase in mature mushroom spines within the left striatum suggested enhanced DRD1-MSN function in KO mice (Supplementary information, Fig. S5e), while the right striatum exhibited significantly reduced long-thin spine volume (Supplementary information, Fig. S5f). For DRD2-MSNs, density and volume of total spine, as well as those of the four specific spine types, remained consistent in the left and right striatum of WT and KO mice (Fig. 5g, h; Supplementary information, Fig. S5d, g, h).

**Fig. 6:**
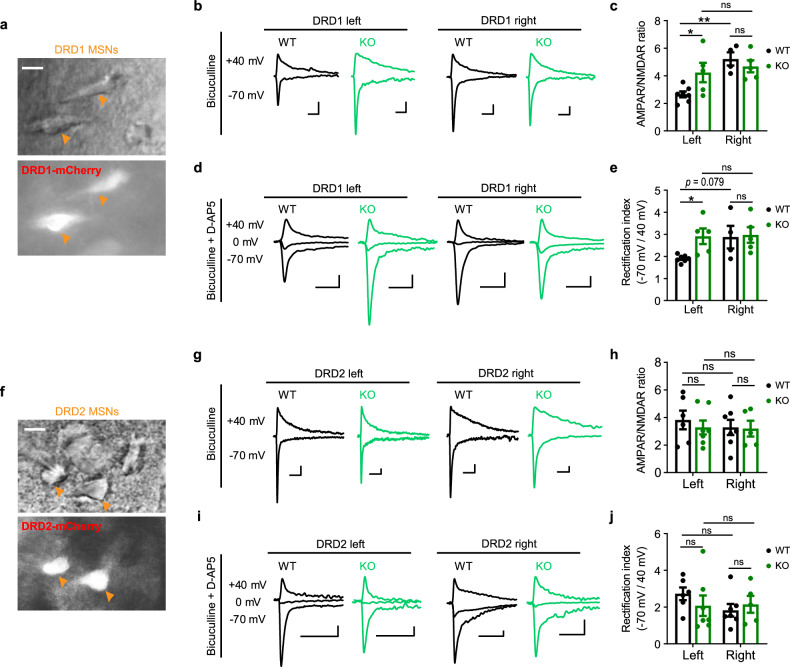
SH3RF2 deficiency induces AMPAR dysfunction of DRD1-MSNs in the left striatum. **a**, **f** Representative images showing the DRD1-MSNs (**a**) and DRD2-MSNs (**f**) labeled with tdTomato (arrowhead). Scale bars, 10 μm. **b**, **g** Typical EPSCs of DRD1-MSNs (**b**) and DRD2-MSNs (**g**) recorded at the holding potential of 40 mV and –70 mV in the presence of bicuculline. Scale bars: 100 ms, 50 pA. **c**, **h** Quantitative results of AMPAR/NMDAR ratio of DRD1-MSNs (**c**) and DRD2-MSNs (**h**). NMDAR EPSCs were measured at 50 ms post stimulation. Two-way ANOVA with Sidak’s multiple comparisons test. **d**, **i** Typical AMPAR EPSCs of DRD1-MSNs (**d**) and DRD2-MSNs (**i**) recorded at the holding potential of –70 mV, 0 mV, and 40 mV in the presence of D-AP5 and bicuculline. Scale bars: 100 ms, 50 pA. **e**, **j** Quantitative results of AMPAR rectification index of DRD1-MSNs (**e**) and DRD2-MSNs (**j**). For DRD1-MSNs, WT: 12 (left) and 7 (right) cells from total 7 mice; KO: 8 (left) and 10 (right) cells from total 5 mice. Each dot represents a mouse (**c**, **e**). For DRD2-MSNs, WT: 8 (left) and 11 (right) cells from total 7 mice; KO: 12 (left) and 8 (right) cells from total 8 mice. Each dot represents a mouse (**h**, **j**). Two-way ANOVA with Sidak’s multiple comparisons test. All data are presented as mean ± SEM; *P < 0.05; **P < 0.01; ns no significance.

To explore the functional implications of *Sh3rf2* deletion in synaptic transmission, we performed whole-cell patch-clamp recordings on DRD1-MSNs and DRD2-MSNs in the DMS of adult mice (Fig. 6a, f). In KO mice, the ratio of AMPAR-mediated evoked excitatory postsynaptic currents (EPSCs) to NMDAR-mediated EPSCs of DRD1-MSNs was significantly increased in the left striatum, mirroring that in the right striatum and disrupting the asymmetry of AMPAR/NMDAR ratio as seen in WT mice (Fig. 6b, c; Supplementary information, Fig. S6a). Furthermore, the rectification index, determined by the ratio of AMPAR-mediated EPSCs at –70 mV to those at +40 mV after NMDAR blockade by D-AP5, was also significantly increased in the left striatum but not in the right striatum of KO mice (Fig. 6d, e; Supplementary information, Fig. S6b). However, there were no significant differences in AMPAR/NMDAR ratio or AMPAR rectification index of DRD2-MSNs between the left and right striatum, and neither metric was altered by *Sh3rf2* deletion (Fig. 6g–j; Supplementary information, Fig. S6c, d).

AMPARs are tetrameric glutamate-gated ion channels composed of a combination of GluR1–GluR4 subunits. GluR2-lacking AMPARs (primarily GluR1 homomers) demonstrate calcium permeability with elevated conductance and strong inward rectification, consequently enhancing synaptic transmission.^37,40^ The increase of rectification index therefore implied the pathological hyperactivity of DRD1-MSNs in the left striatum of *Sh3rf2* KO mice.

### Elevated activity in the left striatum of DRD1-MSNs contributes to autism-like behavior in *Sh3rf2* KO mice

**Fig. 7:**
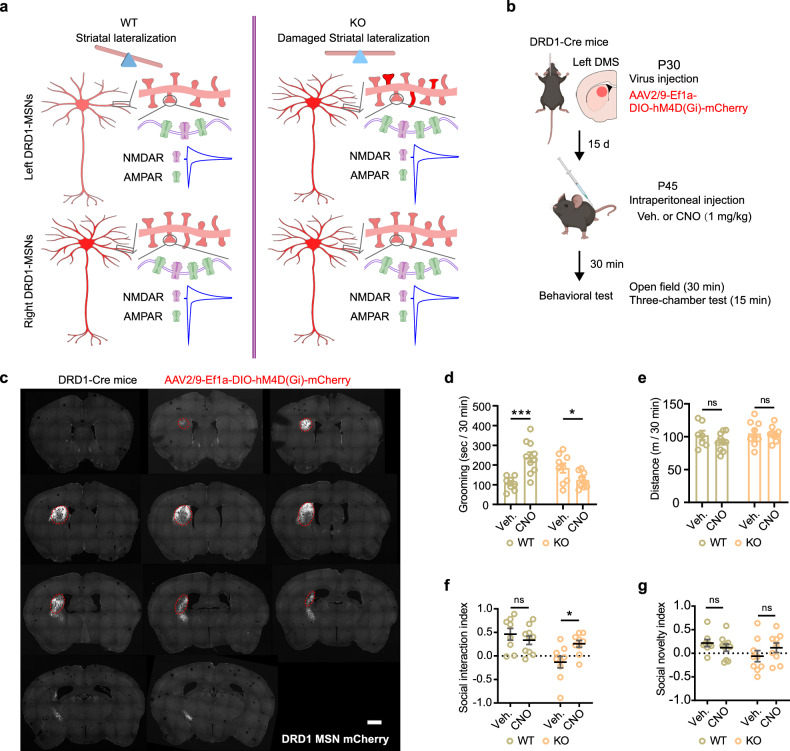
Rescue of autism-like behaviors in *Sh3rf2* deletion mice with DREADD. **a** Schematic depiction of the lateralization of structure and function in striatal DRD1-MSNs. In *Sh3rf2* KO mice, the left striatal DRD1-MSNs exhibit characteristics of the right striatal DRD1-MSNs, resulting in a disruption of striatal lateralization. **b** Schematic diagram of rescue strategy with DREADD and the behavior test paradigm. AAV2/9-Ef1α-DIO-hM4D(Gi)-mCherry was injected into the left DMS of Drd1a-Cre mice. **c** Injection site of AAV in the striatum. Scale bar, 500 μm. **d** Quantitative results of time spent in grooming by WT and KO mice treated with vehicle (Veh) or CNO in open field within 30 min. Inhibition of DRD1-MSNs in left DMS had opposite effects on grooming in WT and KO mice. **e** Comparable moving distance of WT and KO mice treated with Veh or CNO in open field within 30 min. **f**, **g** Quantitative results of social interaction index (**f**) and social novelty index (**g**) of WT and KO mice treated with Veh or CNO. Three chamber assay. *n* = 8–10 mice for each group and two-way ANOVA with Holm-Sidak’s multiple comparisons test for **d**–**g**. All data are presented as mean ± SEM; *P < 0.05; ***P < 0.001; ns no significance.

The aforementioned findings revealed that the left-sided striatal DRD1-MSNs of *Sh3rf2* KO mice exhibited some right-sided characteristics such as increased dendritic complexity, AMPAR/NMDAR ratio and AMPAR rectification index, thereby disrupting the striatal lateralization (Fig. 7a). We conducted a series of behavioral experiments to substantiate that *Sh3rf2* KO mice display autism-like symptoms, including social deficits and repetitive stereotyped behaviors, while maintaining normal motor capacity and memory (Supplementary information, Fig. S7). Subsequently, we investigated whether the heightened activity of DRD1-MSNs in the left striatum contributes to autism-like behaviors in *Sh3rf2* KO mice.

We inhibited the neuronal activity of DRD1-MSNs in the left striatum using the designer receptors exclusively activated by designer drugs (DREADD).^41^ AAV carrying Cre-dependent hM4D(Gi) was injected into the left DMS, a subregion strongly linked to repetitive stereotyped behavior and partial social behavior,^42–46^ in Drd1a-Cre mice (Fig. 7b, c). Two weeks post virus injection, behavioral experiments were conducted in WT and KO mice following administration of vehicle or clozapine-*N*-oxide (CNO). Repetitive grooming time in CNO-treated KO mice was reduced to normal levels, while to our surprise, CNO treatment significantly induced repetitive grooming in WT mice (Fig. 7d). Inhibition of DRD1-MSNs in the left DMS did not result in diminished motor activity in mice, ruling out the possibility that reduced grooming behavior stemmed from impaired motor ability (Fig. 7e). Moreover, in CNO-treated KO mice, the social interaction was partially rescued, whereas social novelty remained unaltered (Fig. 7f, g). Subsequently, the vehicle-treated mice were subjected to further tests using an alternating paradigm (Supplementary information, Fig. S8a). Similarly, CNO treatment inhibited repetitive grooming and improved social interaction and social novelty in KO mice, with these effects dissipating 2 days later (Supplementary information, Fig. S8b–d). Collectively, these results indicated that enhanced activity of DRD1-MSNs in the left striatum is responsible for autism-like behaviors in *Sh3rf2* deletion mice.

## Discussion

Understanding the structural basis of brain lateralization remains a fundamental yet unresolved question in neuroscience. As emphasized in a recently published review,^47^ investigating the mechanisms underlying the formation of brain lateralization and its link to brain disorders is a crucial and promising scientific pursuit. In this study, we conducted proteomic and phosphoproteomic analyses of the bilateral striatum to uncover the molecular basis of striatal lateralization. Phosphorylation is an exceptionally dynamic modification of proteins, enabling cells to rapidly respond to various extracellular signals, including cytokines, hormones, neurotransmitters, and neurotrophic factors, as well as physical stimuli.^48^ Under basal conditions, the left striatum exhibits a higher abundance of phosphorylated sites compared to the right, corresponding to enrichment of phosphorylation regulatory proteins in the right striatum. This reflects the divergence in phosphorylation regulatory environments essential for supporting striatal lateralization.

Although brain lateralization abnormalities are widely reported in ASD patients, research using animal models has been limited, primarily due to the inherent subtlety of this phenomenon and the resultant difficulty in its detection. We demonstrate that mouse striatal DRD1-MSNs exhibit morphological and functional rightward lateralization mirroring human caudate asymmetry. Strikingly, this asymmetrical organization is disrupted in our *Sh3rf2* deletion mouse model, providing pivotal insights into the mechanisms of striatal lateralization and its pathological associations with ASD-like behaviors. SH3RF2 is highly expressed in striatal MSNs, where it orchestrates a PSD-localized complex with PPP1CC to maintain functional lateralization. We observe significantly higher PPP1CC protein expression in the right striatum of P42 mice. This asymmetry may serve to counterbalance elevated excitability and AMPAR-mediated transmission in right DRD1-MSNs, which would otherwise persistently suppress PP1 activity via DARPP-32-Thr34 phosphorylation.^49^ Increasing PPP1CC abundance provides a reserve of PP1 catalytic capacity, enabling rapid dampening of synaptic strength following DRD1 signaling downregulation — a critical adaptation for maintaining plasticity and homeostasis in hyperactive circuits. Under basal conditions, elevated PPP1CC levels in the right striatum suppress CaMKII-Thr287/286 phosphorylation via SH3RF2-mediated scaffolding. *Sh3rf2* deletion disrupts the critical CaMKII–PPP1CC interaction, causing bilateral hyperphosphorylation of CaMKII-Thr287/286. Interestingly, elevated GluR1-Ser831 phosphorylation emerges selectively in the left striatum, as the richer PPP1CC pool in the right striatum may sustain more stringent phosphorylation regulatory thresholds. By contrast, elevated postsynaptic GluR1 in right DRD1-MSNs likely initiates this compensatory response, and the molecular mechanisms underlying the association between GluR1 accumulation and PPP1CC upregulation remain to be explored.

Another fascinating phenomenon is that the loss of SH3RF2 primarily affects DRD1-MSNs, with minimal impact on DRD2-MSNs. This reflects the differences in signaling pathways mediated by the two types of dopamine receptors, as well described in the review by Surmeier et al.^50^ In DRD1-MSNs, dopamine signaling promotes the opening of Ca_v_1.3 L-type calcium channels and the surface expression of GluR1, while in DRD2-MSNs, it inhibits these processes. Therefore, the impact of CaMKII activation caused by *Sh3rf2* deletion in DRD2-MSNs might be counteracted by dopamine signaling. It is not unprecedented that knockout of the same gene affects the two types of MSNs differently. Previous research has shown that *Shank3B* deficiency leads to significant synaptic defects in DRD2-MSNs, but not in DRD1-MSNs.^46^

Interestingly, the ASD-like behaviors in *Sh3rf2* deletion mice can be rescued by inhibiting the activity of DRD1-MSNs in the left striatum. In addition to our findings, another study has found that inhibition of the excitatory neurons in the left medial prefrontal cortex (mPFC) ameliorated social deficits and repetitive behaviors in Purkinje cell-*Tsc1* mutant mice.^51^ These findings raise the possibility that differential hemispheric susceptibility to ASD-related genetic perturbation could contribute to the pervasive asymmetrical changes observed in the brains of autism patients. Notably, we found that inhibition of the left striatal DRD1-MSNs in WT mice induced significant excessive grooming behavior, implying that disruption of coordination between the direct and indirect pathways in the striatum or between the hemispheres may be associated with ASD-like behaviors.

While our study has uncovered the crucial role of the SH3RF2/CaMKII/PPP1CC protein complex in maintaining striatal lateralization, several limitations remain. Firstly, the phosphorylation levels of proteins in the striatum are influenced by at least two factors: the internal content of proteins involved in phosphorylation regulation (e.g., PP1) and the sensitivity of neurons to external stimuli (e.g., the amount of neurotransmitter receptors on the membrane). These two factors are contradictory yet interdependent, making it challenging to quantify the intensity of phosphorylation regulation in bilateral striatum. Secondly, similar to many studies in zebrafish models, while regulatory elements affecting lateralization have been identified, the driving elements that generate lateralization remain unknown. Specifically, in our study, we are currently unable to ascertain why there is a difference in the AMPAR content at the postsynaptic membrane between the bilateral striatum in normal mice. Future studies will warrant systematic dissection of the origin of asymmetric postsynaptic GluR1 enrichment — the fundamental driver of striatal lateralization — by exploring how synaptic inputs, receptor trafficking, and activity-dependent signals converge to establish this AMPAR asymmetry and its functional integration with PPP1CC-mediated homeostasis.

In summary, we have not only identified a new ASD-associated protein complex but also elucidated its essential role in maintaining brain functional lateralization from both cellular and molecular perspectives. Insights gained by revealing SH3RF2-mediated molecular mechanisms underlying striatal asymmetry disruption and ASD-like behaviors would hold potential to reshape the perceptions of brain function and neurodevelopmental disorders.

## Materials and methods

### Animals

*Sh3rf2* KO mice have been described previously.^21^ *Sh3rf2-3*× *flag* knockin mice were generated and kept by Dr. Zhiheng Xu’s group. Drd1a-Cre and Drd2-Cre mice were gifts from Dr. Minmin Luo (National Institute of Biological Sciences, Beijing), and Ai14 mice were gifts from Dr. Weixiang Guo (Institute of Genetics and Developmental Biology, Chinese Academy of Sciences). All mice were maintained on C57BL/6J background. C57BL/6J WT mice were used as controls for *Sh3rf2* KO mice. All mice were housed in a 21–24 °C and 40%–60% humidity facility with free access to food and water. All animal experiments were approved by the Institutional Animal Care and Use Committee (issue# AP2022053) at Institute of Genetics and Developmental Biology, Chinese Academy of Sciences.

### Proteomic and phosphoproteomic analyses

The proteomic and phosphoproteomic analyses of bilateral striatum were conducted at the proteomics facility of the Institute of Genetics and Developmental Biology, Chinese Academy of Sciences. Tissue collection: it is imperative to meticulously control conditions during sample collection to minimize external interferences. All samples were collected between 4:00 p.m. and 5:00 p.m. 42-day-old male C57BL/6J mice, maintained in a calm state, were swiftly decapitated, and the bilateral striatum was rapidly isolated on ice. For the first batch of mice, the left striatum was collected first, followed by the right striatum, and for the second batch of mice, the order was reversed. Subsequently, the left and right striatum from the first batch were mixed with those from the second batch to eliminate any effects of dissection timing on protein phosphorylation. Protein extraction: after the striatal tissue was removed from liquid nitrogen, sodium deoxycholate (SDC) lysis buffer (4% SDC, 100 mM Tris-HCl, pH 8.5) was added at a ratio of 20 μL per mg of tissue, and the tissue was quickly homogenized on ice with a handheld grinder. Then, the homogenate was immediately boiled for 10 min and centrifuged for 10 min to collect the supernatant. After determination of protein concentration using a BCA protein assay kit, all samples were stored at –80 °C until use. The detailed experimental procedures, excluding tissue collection and protein extraction, have been thoroughly described in the published article,^26^ and are therefore not repeated here. Data analysis: Proteins and phosphorylation sites detected exclusively in one side (in at least two samples) and those with a fold change > 1.25 or < 0.8 and *P*-value < 0.05 (paired *t*-test) were deemed significantly differentially expressed. The GO analysis and GSEA were conducted using the clusterProfiler package^52^ in R. The final results were visualized using the ggplot2 package^53^ and the enrichplot package.

### Detection of SH3RF2-interacting proteins via mass spectrometry

The striatal tissues from P42 *Sh3rf2-3× flag* male mice and their WT littermates were used for co-IP. For each sample, 200 μg of proteins were incubated with 20 μL Anti-FLAG M2 affinity gel (Sigma) at 4 °C overnight. Then proteins were separated by 10% SDS-PAGE. The proteins in gel were treated with 10 mM DTT at 37 °C for 1 h and alkylated with 55 mM iodoacetamide at room temperature (RT) for 1 h in dark, and digested with trypsin in 25 mM ammonium bicarbonate at 37 °C overnight. Peptides were extracted from gel by sonication with stripping buffer (5% trifluoroacetic acid and 50% acetonitrile). The liquid was dried by SpeedVac, and peptides were desalted by StageTips. The resuspended peptides were analyzed by LTQ Orbitrap Elite mass spectrometer coupled online to an Easy-nLC 1000 in the data-dependent mode. The peptides were separated by reverse phase LC with a 75 μm (ID) × 250 mm (length) analytical column packed with C18 particles of 5 µm diameter. All mass spectrometry measurements were performed in the positive ion mode. Precursor ions were measured in the Orbitrap analyzer at 240,000 resolutions (at 400 m/z) and a target value of 1 × 10⁶ ions. The 20 most intense ions from each mass spectrometry scan were isolated, fragmented, and measured in the linear ion trap. The CID normalized collision energy was set to 35. The data was analyzed using a pre-release version of Thermo Scientific Proteome Discoverer software version 1.4. The proteome sequences for *Mus musculus* from UniProt were used for the database searching. The proteins only detected in *Sh3rf2-3× flag* mice (Unique) or whose PSMs (peptide-spectrum matches) in *Sh3rf2-3× flag* mice were more than twice as much as in control mice (fold change ≥ 2) were defined as SH3RF2-interacting proteins. The PPI network analysis was performed on STRING (https://cn.string-db.org/)^54^ combined with Cytoscape (3.7.0).^55^ The GO analysis and KEGG analysis were performed on DAVID (https://david.ncifcrf.gov/home.jsp).^56^

### Western blot

The prepared tissue lysates were mixed with 5× SDS gel loading buffer (250 mM Tris-HCl, pH 6.8, 50% glycerol, 10% SDS, 0.1% bromophenol blue, 12.5% 2-mercaptoethanol) (5:1) and heated at 95 °C for 5 min. The samples were separated by SDS-PAGE and then transferred to NC blotting membrane (GE Whatman). After blocking with 5% skim milk in TBST (20 mM Tris-HCl, pH 8.0, 0.15 M NaCl, 0.05% Tween-20) at RT for 1 h, membranes were incubated with primary antibodies at 4 °C overnight. After washing with TBST for 10 min three times, membranes were incubated with HRP-conjugated secondary antibodies at RT for 1–2 h, followed by washing with TBST for 10 min three times. Then the membranes were incubated with chemiluminescence substrate, and signals were detected using an automatic chemiluminescence detector.

### Subcellular fractionation and PSD isolation

Subcellular fractionation and PSD isolation has been described previously.^57^ Left and right striatum of adult mice was homogenized in ice-cold sucrose buffer (0.32 M sucrose, 10 mM Tris-HCl, pH 7.4, 1 mM EDTA, pH 8.0, 1 mM EGTA, pH 8.0) containing freshly added protease inhibitors and phosphatase inhibitors (Pierce^TM^ protease and phosphatase inhibitor mini tablets, Thermo Scientific), and then centrifuged at 1000× *g* at 4 °C for 10 min to remove the pelleted nuclear fraction and tissue debris (P1). The supernatant (S1) was centrifuged at 10,000× *g* at 4 °C for 15 min to yield the crude synaptosomal pellet (P2). The supernatant (S2) was then centrifuged at 100,000× *g* at 4 °C for 60 min to separate cytoplasmic proteins (S3) and intracellular light membrane fraction (P3). The P2 was subsequently resuspended in 120 μL sucrose buffer, and mixed with 8 volumes of buffer II (0.5% Triton X-100, 10 mM Tris-HCl, pH 7.4, 1 mM EDTA, pH 8.0, 1 mM EGTA, pH 8.0) containing freshly added protease inhibitors and phosphatase inhibitors. The mixture was homogenized again and mixed for 30 min at 4 °C. The lysate was centrifuged at 32,000× *g* at 4 °C for 30 min in a TL-100 tabletop ultracentrifuge (Beckman). The resulting pellet containing insoluble PSD proteins was considered as the postsynaptic membrane component, and the supernatant (T×S) contains soluble proteins not tightly bound to the PSD. The S3 and T×S fractions were further concentrated by adding 8 volumes of 100% acetone and incubated at 20 °C overnight, and then centrifuged at 3000× *g* at 4 °C for 15 min. The precipitated proteins were dried at RT for 15 min. All pellets were dissolved in TE buffer (100 mM Tris-HCl, pH 8.0, 10 mM EDTA, pH 8.0) containing 1% SDS. Lastly, the lysates were mixed with 5× SDS gel loading buffer (5:1) and heated at 95 °C for 5 min. The samples were subjected to western blot later. Antibodies used here can be found in the antibody list (Supplementary information, Table S5).

### Co-IP

Co-IP was performed using Pierce^TM^ Protein A/G Magnetic Beads (Thermo Scientific) or Dynabeads^TM^ Protein G Magnetic Beads (Invitrogen) according to the manufacturer’s instructions with minor modifications. Briefly, striatum tissues were digested with endogenous lysis buffer (20 mM Tris-HCl, pH 7.4, 50 mM NaCl, 0.5% NP-40, 10 mM HEPES, pH 7.4, 0.5 mM EDTA, pH 8.0) containing freshly added protease inhibitors and phosphatase inhibitors. Primary antibodies (2 μg) were incubated with beads (25 μL) at 4 °C for 2 h, followed by incubation with tissue lysates (300–500 μL) at 4 °C overnight. Then, beads were collected using a magnetic stand and washed twice with TBST, and once with purified water. Immunoprecipitates were stripped off by boiling in 2× SDS gel loading buffer and subjected to western blot later.

### Immunofluorescence staining

For mouse brain sections, transcardially perfused mouse brains were fixed with 4% paraformaldehyde (PFA) at 4 °C for 24 h, and dehydrated in 30% sucrose at 4 °C for 48 h. Then the brains were embedded in optimal cutting temperature compound (O.C.T. Compound, SAKURA, REF4583, USA). Sections (40 μm thick) were prepared with freezing microtome (CM 1950, Leica, Germany). For cultured neurons, the neurons were fixed with 4% PFA at RT for 10 min. Fixed sections or neurons were blocked with blocking buffer (PBS + 10% FBS + 3% BSA + 0.2% Triton X-100) at RT for 1 h and incubated with primary antibodies at 4 °C overnight. After washing with PBST (PBS + 0.2% Triton X-100) for 10 min three times, sections or neurons were incubated with appropriate fluorescent secondary antibodies (1:2000; Invitrogen) and DAPI (Cell Signaling Technology, Cat# 4083s) at RT for 1 h, followed by PBST washing for 10 min three times. Fluorescent images were captured using a confocal laser-scanning microscope (Carl Zeiss, LSM800) and analyzed with ZEN3.1 and ImageJ. Antibodies used here can be found in the antibody list (Supplementary information, Table S5).

### Striatal neuron culture

The lateral ganglionic eminence of E17.5 mouse embryos was dissected and dissociated with trypsin-EDTA (Gibco) at 37 °C for 15 min. Then, trypsin-EDTA was removed and 1 mL cell culture medium (DMEM/F12 (Gibco) + 10% FBS (Excell)) was added to terminate the digestion, and repeated 3 times. 1 mL cell culture medium was added and the tissue was gently blown into a single cell. The digested tissue stood for 2 min to allow undigested tissue and vascular membrane to settle. The supernatant containing single cells was seeded into the poly-L-lysine (Sigma) coated 24-well plate at 5 × 10^4^ cells/well. Neurons were cultured in the incubator with 5% CO_2_ and saturated humidity at 37 °C for 4 h. Then, the medium was changed to the serum-free neuronal medium (Neurobasal (Gibco) + 1% GlutaMAX^TM^ (Gibco) + 2% B27 (Gibco)), and was half replaced with fresh medium every 3 days. Neuronal transfection was performed using Lipofectamine 2000 (Invitrogen) according to the manufacturer’s instructions. Briefly, 1 μg plasmid was mixed with 0.5 μL Lipofectamine 2000 in 25 μL Neurobasal medium, incubated for 20 min, and then added to the neurons. After 1 h, the medium containing plasmid was replaced with fresh neuronal medium, and the neurons continued to grow in the incubator.

### Morphological characterization of dendrites and spines

To sparsely label DRD1 and DRD2 MSNs, 60 nL AAV2/1-Ef1α-DIO-mCherry virus (OBiO Technology, Shanghai) with the titer of 1 × 10^13^ GC/mL was injected into bilateral thalamic reticular nucleus (AP: –0.6 mm; ML: ±1.6 mm; DV: –3.65 mm, referred to Paxinos and Franklin’s the Mouse Brain in Stereotaxic Coordinates, third edition,^58^ with stereotaxic apparatus (RWD, China)) of Drd1a-Cre or Drd2-Cre mice at the age of 5 weeks. Three weeks later, the mice were euthanized for immunostaining. mCherry signals were amplified with anti-mCherry antibody. mCherry-labeled MSNs in dorsomedial striatum were captured using a confocal laser-scanning microscope, and the images were processed with Imaris software (7.6.0).

### Electrophysiology

Brain slice preparation: coronal brain slices (300 μm) containing the striatum were prepared and processed as follows. Briefly, adult mice (8–12 weeks) were anesthetized with avertin and perfused transcardially with 20 mL ice-cold cutting solution (110 mM C_5_H_14_NClO, 2.5 mM KCl, 25 mM NaHCO_3_, 1.3 mM NaH_2_PO_4_, 7 mM MgCl_2_, 0.5 mM CaCl_2_, and 25 mM glucose) adjusted to pH 7.3–7.4, saturated with 95% O_2_ and 5% CO_2_. The brain was quickly removed and sliced on a vibratome (Leica VT1200s, Wetzlar, Germany) containing ice-cold cutting solution. The slices were incubated for 30 min at 37 °C and then maintained at RT (22–25 °C) in the artificial cerebro-spinal fluid (aCSF) (125 mM NaCl, 2.5 mM KCl, 25 mM NaHCO_3_, 1.3 mM NaH_2_PO_4_, 1.3 mM MgCl_2_, 2.5 mM CaCl_2_, and 25 mM glucose) adjusted to pH 7.3–7.4, saturated with 95% O_2_ and 5% CO_2_, and then kept at RT for recording.

Electrophysiological recordings: the slice was placed in a recording chamber and constantly perfused with oxygenated aCSF at 25 °C (TC-324B, Warner Instruments, USA) at a rate of 1.5–2.0 mL/min. Cells were visualized using IR-DIC optics on an inverted Olympus BX51WI microscope. DRD1/DRD2 MSNs were identified by the morphology and strong fluorescence of tdTomato. Whole-cell recordings were performed with MultiClamp Amplifier 700B and Digidata Digitizer 1550B (Molecular Devices Corporation, Sunnyvale, CA, USA). The signals were acquired at 20 KHz and filtered at 2 KHz. The access series resistance of the neurons used for analysis was < 20 mΩ. Cells were voltage-clamped at –70 mV with 4–6 mΩ patch pipettes. The internal solution contained: 145 mM CsCl, 8 mM NaCl, 1 mM MgCl_2_, 10 mM HEPES, 2 mM Mg_2_ATP, and 0.2 mM Na_2_GTP, pH 7.2, adjusted with CsOH. AMPAR- and NMDAR-mediated synaptic current ratio (AMPAR/NMDAR ratio) was recorded in the presence of 10 μM bicuculline at holding potentials of –70 mV and +40 mV, respectively. The peak current at –70 mV was considered fully mediated by AMPARs, whereas NMDAR-mediated responses were recorded at +40 mV and measured 50 ms after the stimulus to avoid contamination of AMPAR currents. The AMPAR/NMDAR ratio was calculated by dividing the average peak amplitude of AMPAR-mediated EPSCs by that of NMDAR-mediated EPSCs. The pure AMPAR-EPSCs were recorded in the presence of 10 μM bicuculline and 50 μM D-AP5. Rectification index values were calculated as the ratio of AMPAR-mediated EPSCs at –70 mV to that at +40 mV. All EPSCs used for analysis were averaged from 6 consecutive traces with a stimulus interval of 10 s. Data were analyzed using Clampfit 10 software.

### Rescue of autism-like behaviors with chemogenetics

150 nL AAV2/9-Ef1α-DIO-hM4Di-mCherry virus (OBiO Technology, Shanghai) with the titer of 1 × 10^13^ GC/mL was injected into the left dorsomedial striatum (AP: +0.26 mm; ML: +1.5 mm; DV: –2.9 mm) of Drd1a-Cre mice on postnatal day 30. Fifteen days later, the mice were subjected to behavioral tests. To inhibit the neuron activities, the mice were intraperitoneally injected with CNO (Sigma) at the dose of 1 mg/kg.^41^ The behavioral tests were conducted 30 min later.^59^

### Behavioral experiments

All behavioral tests, unless otherwise specified, were performed as described previously with minor modifications.^21^ All behavioral tests were conducted between 14:00 and 18:00. All mice subjected to behavioral tests were 6–7-week-old male mice except the mice in Supplementary information, Fig. S7c, f (6–7-week-old female mice). More details of the behavioral experiments can be found in Supplementary information, Data S1.

### Quantification and statistical analyses

Data were analyzed using GraphPad Prism (v.8.0.2). All summary data are presented in the text as mean ± SEM. The statistical methods of each figure can be found in figure legends. Normally-distributed data were analyzed using Student’s *t*-test (for 2 groups) or two-way ANOVA (for > 2 groups), unless otherwise specified. Non-normally distributed data were analyzed using the Mann-Whitney U test (for 2 groups). Multiple comparison adjusted *P* values were computed using Sidak’s multiple comparison test or Tukey post-hoc test. *P* < 0.05 was considered statistically significant.

## Supplementary information

Supplementary information, Figure S1

Supplementary information, Figure S2

Supplementary information, Figure S3

Supplementary information, Figure S4

Supplementary information, Figure S5

Supplementary information, Figure S6

Supplementary information, Figure S7

Supplementary information, Figure S8

Supplementary information, Table S1

Supplementary information, Table S2

Supplementary information, Table S3

Supplementary information, Table S4

Supplementary information, Table S5

Supplementary information, Data S1

## Data Availability

Raw proteomic data and raw phosphoproteomic data have been deposited to the ProteomeXchange Consortium via the PRIDE^60^ partner repository with the dataset identifier PXD056740 (Token: kODpkbS1vaJS). Further information and requests for resources and reagents should be addressed to and will be fulfilled by the lead contact Zhiheng Xu (zhxu@genetics.ac.cn).
