# Supplementary material for: Autism-related proteins form a complex to maintain the striatal asymmetry in mice
Source: Cell Res. 2025 Sep 2;35(10):762–74. doi: 10.1038/s41422-025-01174-9 (PMC12485048; doi:10.1038/s41422-025-01174-9)
Supplement: Supplementary file 1 — Supplementary information, Figure S1 [file 41422_2025_1174_MOESM1_ESM.pdf]

Supplementary Figure 1

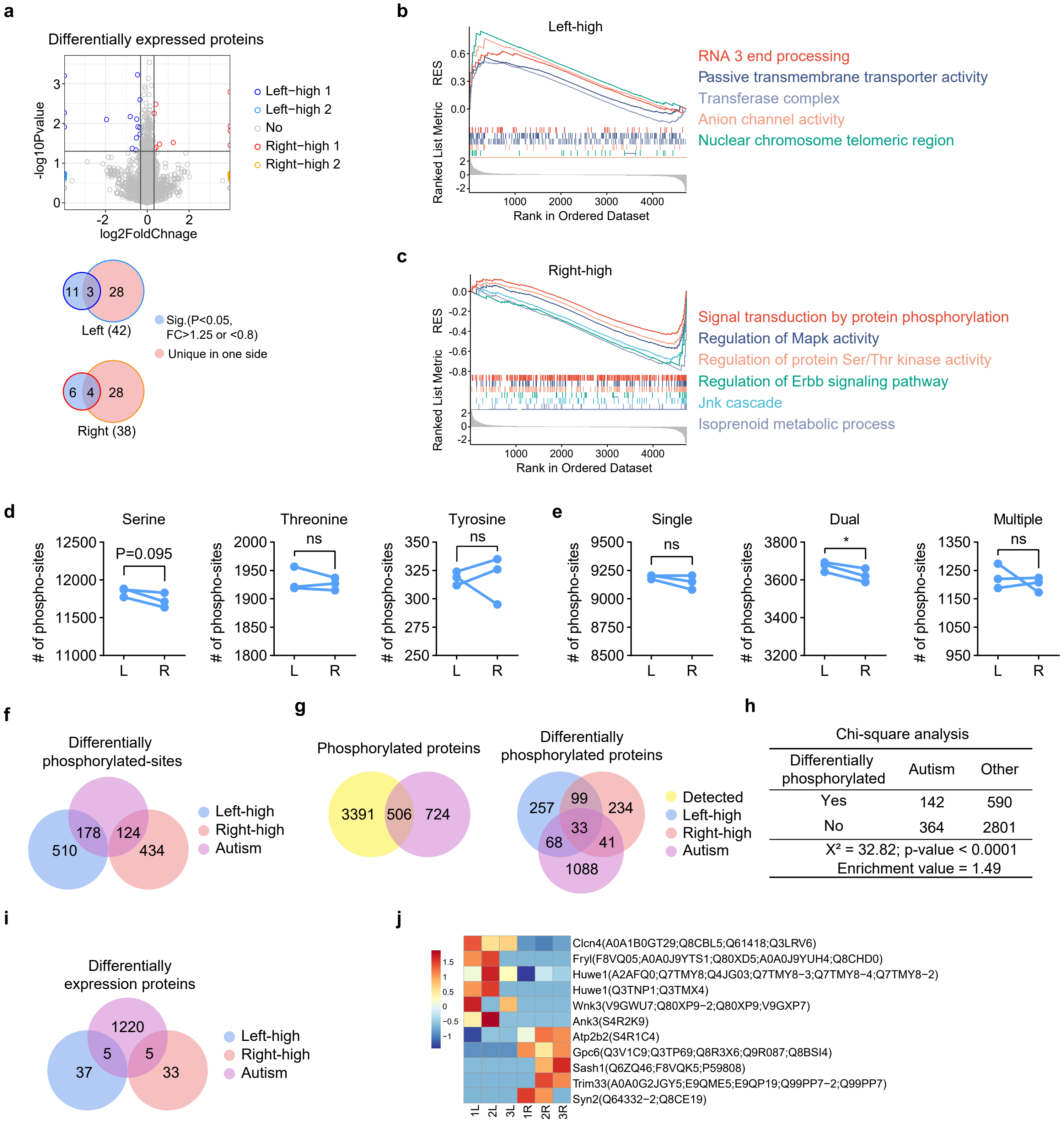

Asymmetries in protein expression and phosphorylation in bilateral striatum reveled by proteomics and phosphoproteomics. a Volcano plot and Venn diagram displayed the number of proteins with significantly differential expression between the left and right striatum. These sites and proteins exhibit statistical significance (p value < 0.05, Fold Change > 1.25 or < 0.8) between two sides or are detected exclusively on one side (in at least two samples from the same side). **b**, **c** The gene set enrichment analysis of the differentially expressed proteins between left and right striatum. The representative enriched functional terms of left (**b**) and right (**c**) striatum are presented. **d** Statistical results of the number of phosphorylated serine, threonine and tyrosine sites detected by phosphoproteomics in the bilateral striatum. Paired t test. **e** Statistical results of the number of monophosphorylated, bisphosphorylated and mutiphosphorylated peptide segments detected by phosphoproteomics in the bilateral striatum. Paired t test. **f** Venn diagram illustrates the number of sites with significantly differential phosphorylation levels of autism-related proteins in the left and right striatum. **g** Venn diagram illustrates the number of autism-related proteins with significantly differential phosphorylation sites in the left and right striatum. **h** Chi-square analysis showing significant enrichment of differentially phosphorylated proteins among ASD-related genes. **i** Venn diagram illustrates the number of autism-related proteins with significantly differential expression in the left and right striatum. **j** Heatmap illustrates the autism-related proteins with significantly differential expression in the left and right striatum. *p < 0.05; ns: no significance.
