# Supplementary material for: Autism-related proteins form a complex to maintain the striatal asymmetry in mice
Source: Cell Res. 2025 Sep 2;35(10):762–74. doi: 10.1038/s41422-025-01174-9 (PMC12485048; doi:10.1038/s41422-025-01174-9)
Supplement: Supplementary file 2 — Supplementary information, Figure S2 [file 41422_2025_1174_MOESM2_ESM.pdf]

Supplementary Figure 2

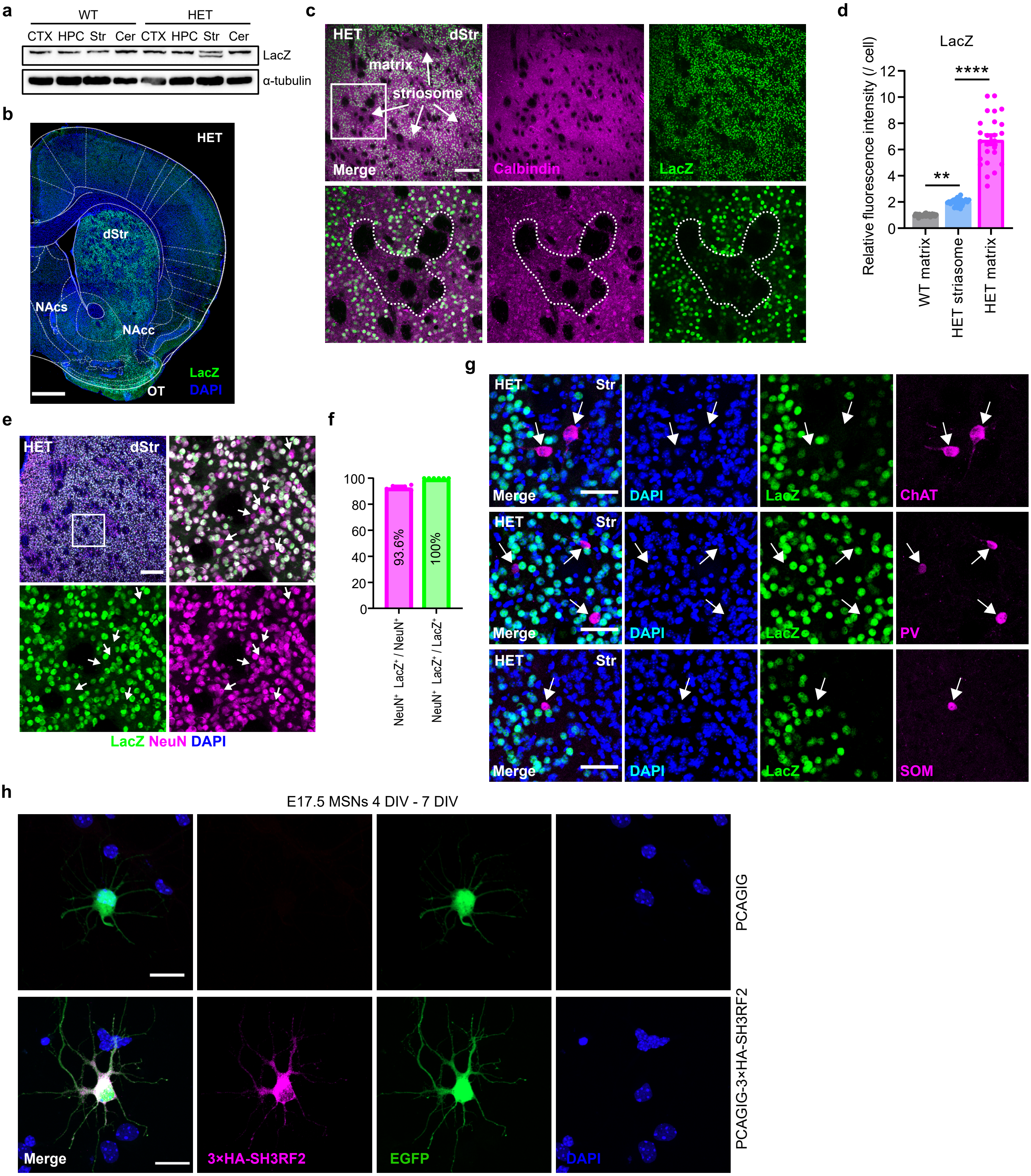

Spatial expression profile of SH3RF2 in the striatum. a Western blot results showing that LacZ was specifically detected in the striatum of HET mouse. **b** Coronal brain slice of HET mice were immunostained with LacZ antibody and DAPI. dStr: dorsal striatum; NAcc: nucleus accumbens core; NAcs: nucleus accumbens shell; OT: olfactory tubercle. Scale bar: 1000 μm. **c** Representative images showing that Sh3rf2 was highly expressed in the matrix of striatum. Calbindin: a marker of matrix. Zoom in area of the white box, dotted area and arrows represent the striosomes. Scale bar: 200 μm. **d** Quantitative results of relative fluorescence intensity of LacZ signal of each cell in striatal matrix and striosomes. WT mice were used as negative control. n = 25 cells for each group. One-way ANOVA with Tukey's multiple comparisons test. **e** HET mice were immunostained with LacZ antibody, NeuN antibody and DAPI. Zoom in the area of the white box. White arrow: neuron without LacZ. Scale bar: 200 μm. **f** Quantitative results of the co-localization of LacZ with NeuN in dorsal striatum. n = 6 (2 slices, 3 regions per slice). **g** Striatal coronal sections of HET mice were immunostained with LacZ antibody and the antibodies for interneurons. White arrow: interneuron without LacZ signal. Scale bar: 50 μm. **h** Representative neuron expressing 3×HA-SH3RF2 was immunostained with HA and GFP antibodies and DAPI. Scale bars: 20 μm. All data are presented as mean ± SEM; $^{**}p < 0.01$, $^{****}p < 0.0001$; ns: no significance.
