# Supplementary material for: Autism-related proteins form a complex to maintain the striatal asymmetry in mice
Source: Cell Res. 2025 Sep 2;35(10):762–74. doi: 10.1038/s41422-025-01174-9 (PMC12485048; doi:10.1038/s41422-025-01174-9)
Supplement: Supplementary file 3 — Supplementary information, Figure S3 [file 41422_2025_1174_MOESM3_ESM.pdf]

Supplementary Figure 3

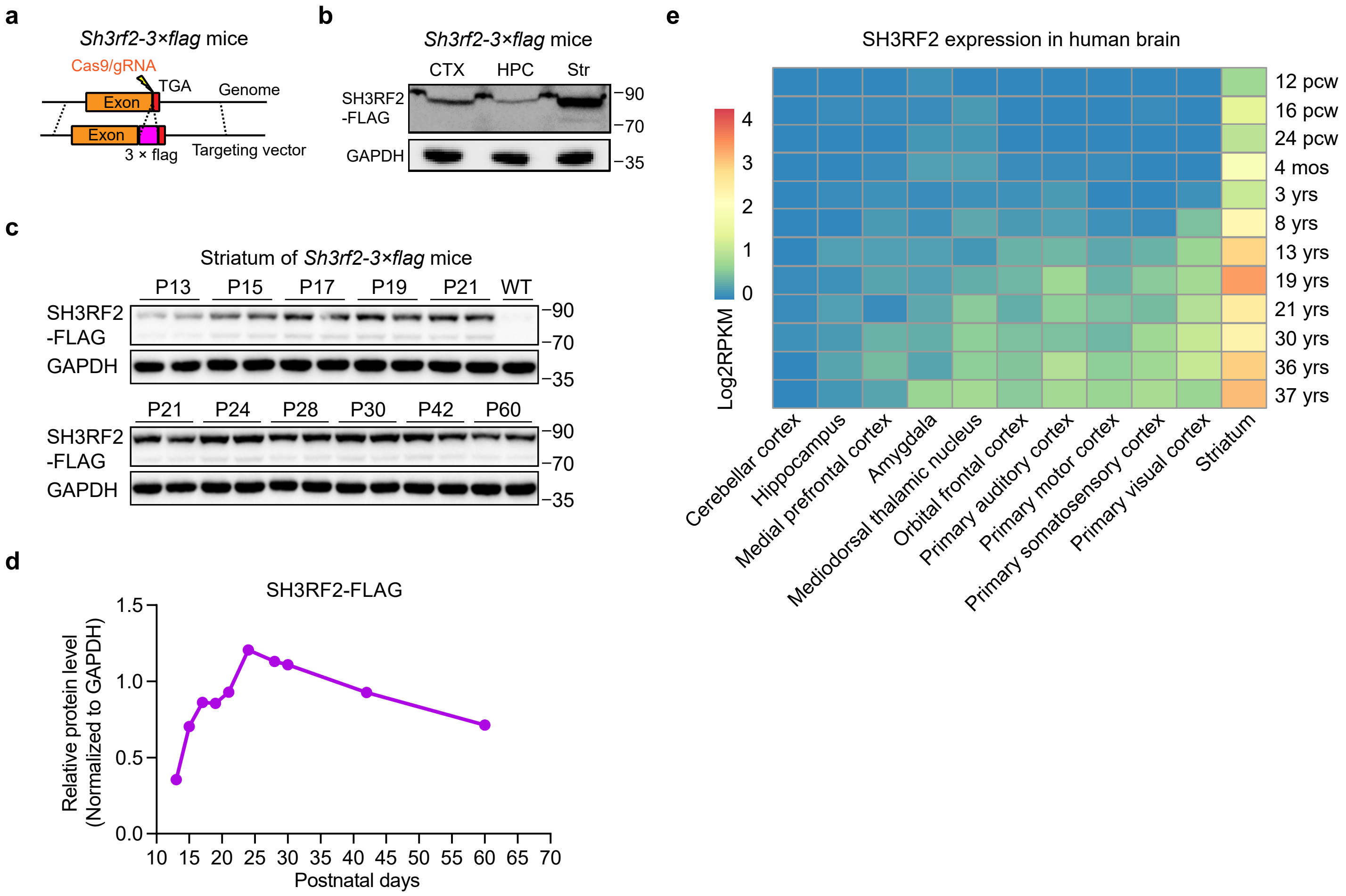

Temporal expression profile of SH3RF2 in the striatum. **a** Schematic diagram of Sh3rf2-3×flag knockin strategy using CRISPR/Cas9. Three consecutive sequences of flag were inserted before the stop codon of Sh3rf2. **b** Western blot results showing that SH3RF2 was mainly detected in the striatum of Sh3rf2-3×flag mice. **c**, **d** Western blots and quantitative result displayed the expression levels of SH3RF2 in the striatum over time. All data are presented as mean ± SEM. **e** Expression levels of SH3RF2 in different regions of human brain. The data was obtained from BRAINSPAN database. pcw: postconceptional weeks; mos: months old; yrs: years old.
