# Supplementary material for: Autism-related proteins form a complex to maintain the striatal asymmetry in mice
Source: Cell Res. 2025 Sep 2;35(10):762–74. doi: 10.1038/s41422-025-01174-9 (PMC12485048; doi:10.1038/s41422-025-01174-9)
Supplement: Supplementary file 4 — Supplementary information, Figure S4 [file 41422_2025_1174_MOESM4_ESM.pdf]

Supplementary Figure 4

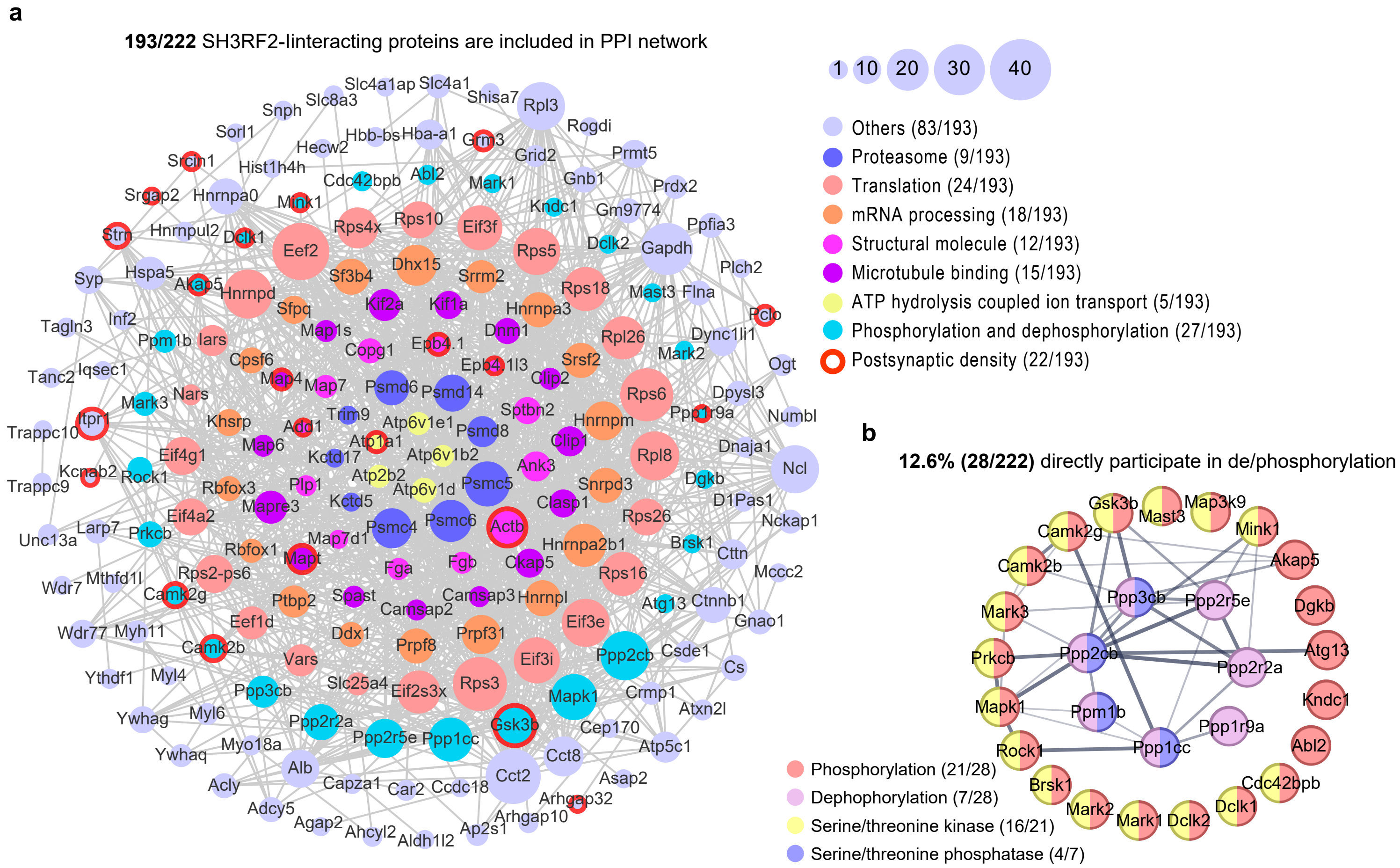

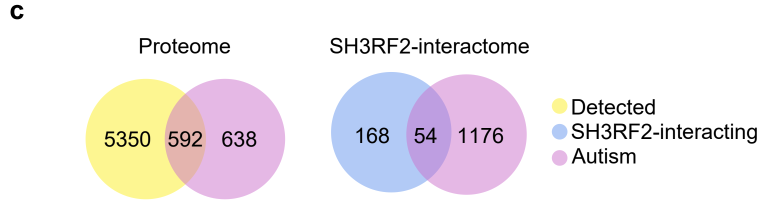

d

Chi-square analysis

SH3RF2-interacting	Autism	Other
Yes	54	168
No	538	5182

$X^2 = 53.02$; p-value < 0.0001
Enrichment value = 2.44

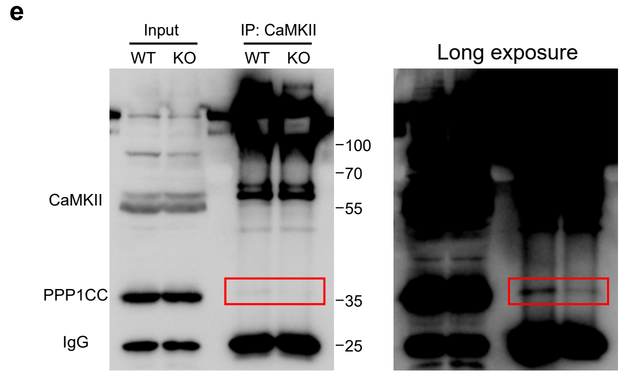

The molecular functions of SH3RF2. **a** Protein-protein interaction (PPI) network of potential SH3RF2-interacting proteins detected in mass spectrum analysis. PPI analysis was performed on STRING (https://cn.string-db.org/). The color of nodes represented the gene categories got from GO analysis. The size of nodes represented the number of nodes connected with the targeted node. Red circle means the protein is localized on postsynaptic density. **b** PPI network of SH3RF2-interacting proteins involved in protein phosphorylation and dephosphorylation. The information of kinases and phosphatases was referred to Uniprot (https://www.uniprot.org/). The thickness of the lines between proteins indicates the confidence index of the interaction. **c** Venn diagram illustrates the number of autism-related proteins among SH3RF2-interacting proteins. **d** Chi-square analysis showing significant enrichment of autism-related proteins among SH3RF2-interacting proteins. **e** Full membrane of the Co-IP assay showed reduced interaction between CaMKII and PP1 in the striatum of KO mice.
