# Supplementary material for: Autism-related proteins form a complex to maintain the striatal asymmetry in mice
Source: Cell Res. 2025 Sep 2;35(10):762–74. doi: 10.1038/s41422-025-01174-9 (PMC12485048; doi:10.1038/s41422-025-01174-9)
Supplement: Supplementary file 5 — Supplementary information, Figure S5 [file 41422_2025_1174_MOESM5_ESM.pdf]

Supplementary Figure 5

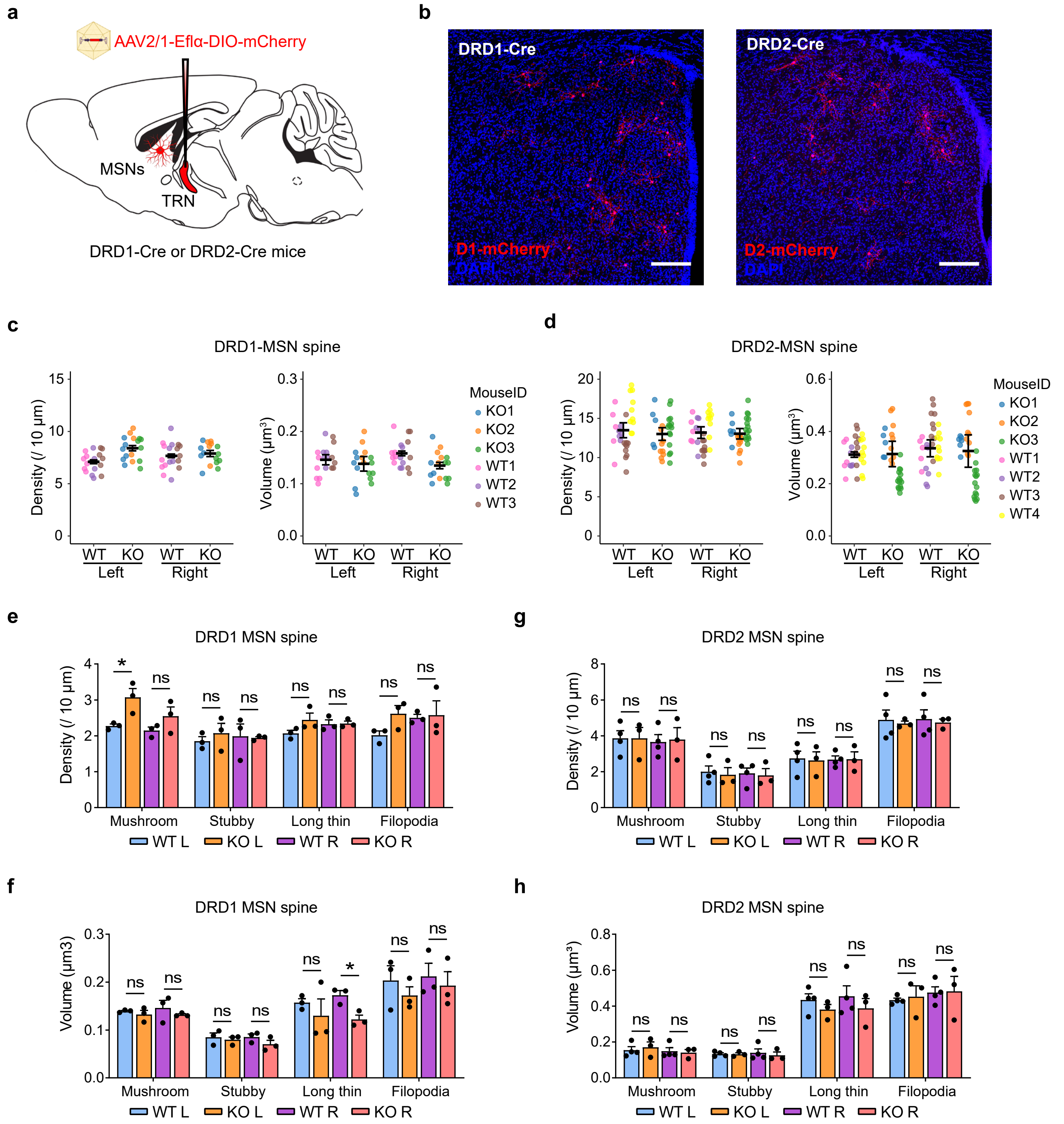

Sparsely label striatal DRD1-MSNs and DRD2-MSNs with AAV. **a** Schematic diagram of sparse labeling strategy. AAV carrying Cre-dependent mCherry coding gene was injected into the thalamic reticular nucleus of DRD1-Cre or DRD2-Cre mice, and the fluorescent signals were amplified with mCherry antibody. **b** Representative images showing the sparse labeling of striatal MSNs in DRD1-Cre and DRD2-Cre mice. Fluorescence signal of mCherry was amplified by immunostaining with anti-mCherry antibody. Scale bar: 200 μm. **c**, **d** Quantitative analysis of the density (left) and volume (right) of DRD1-MSN spines (**c**) and DRD2-MSN spines (**d**). Each dot represents a dendrite, and dots of the same color represent from the same mouse. **e-h** Quantitative analysis showing the density (**e**, **g**) and volume (**f**, **h**) of four types of spine of DRD1-MSNs (**e**, **f**) and DRD2-MSNs (**g**, **h**). n = 3-4 mice for each group, and 5-9 dendrites for each mouse. Each dot represents a mouse. Linear mixed model (genotype × hemisphere + (1|mouse)) with ANOVA and Tukey post-hoc tests. All data are presented as mean ± SEM (mouse level); $*p < 0.05$; ns: no significance.
