# Supplementary material for: Autism-related proteins form a complex to maintain the striatal asymmetry in mice
Source: Cell Res. 2025 Sep 2;35(10):762–74. doi: 10.1038/s41422-025-01174-9 (PMC12485048; doi:10.1038/s41422-025-01174-9)
Supplement: Supplementary file 6 — Supplementary information, Figure S6 [file 41422_2025_1174_MOESM6_ESM.pdf]

Supplementary Figure 6

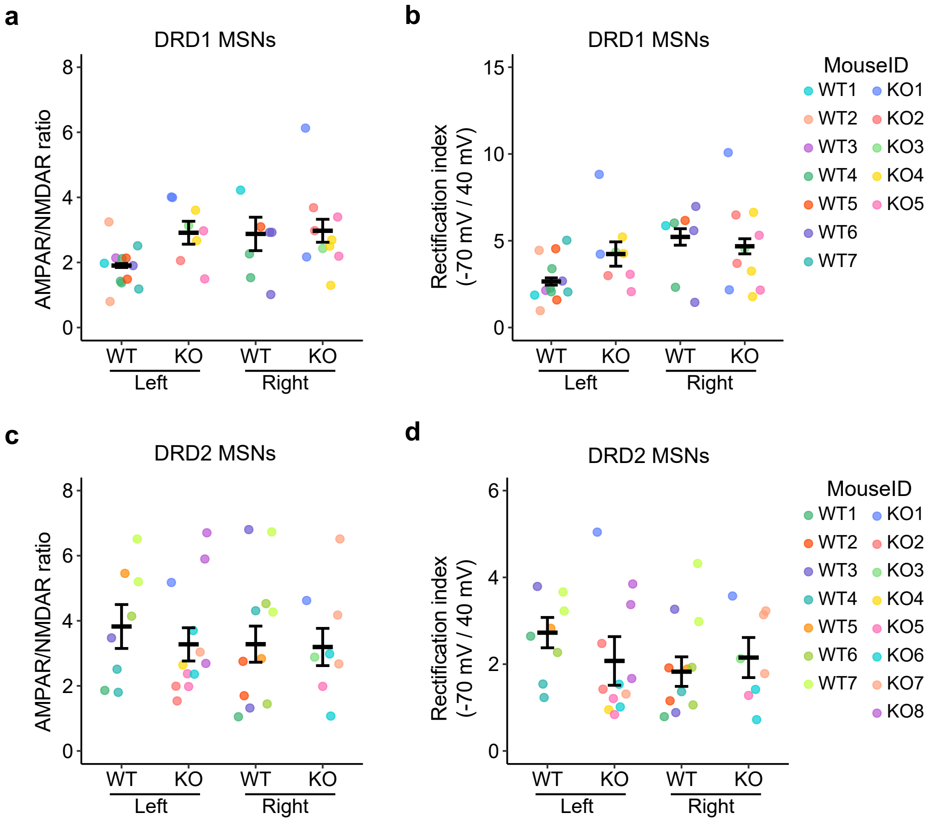

SH3RF2 deficiency induces AMPAR dysfunction of DRD1-MSNs in the left striatum. **a**, **b** Quantitative results of AMPAR/NMDAR ratio (**a**) and rectification index (**b**) of DRD1-MSNs. **c**, **d** Quantitative results of AMPAR/NMDAR ratio (**c**) and rectification index (**d**) of DRD2-MSNs. For DRD1-MSNs, WT: 12 (left) or 7 (right) cells from total 7 mice, KO: 8 (left) or 10 (right) cells from total 5 mice; For DRD2-MSNs, WT: 8 (left) or 11 (right) cells from total 7 mice, KO: 12 (left) or 8 (right) cells from total 8 mice. Each dot represents a neuron, and dots of the same color represent from the same mouse. All data are presented as mean ± SEM (mouse level).
