# Supplementary material for: Autism-related proteins form a complex to maintain the striatal asymmetry in mice
Source: Cell Res. 2025 Sep 2;35(10):762–74. doi: 10.1038/s41422-025-01174-9 (PMC12485048; doi:10.1038/s41422-025-01174-9)
Supplement: Supplementary file 7 — Supplementary information, Figure S7 [file 41422_2025_1174_MOESM7_ESM.pdf]

Supplementary Figure 7

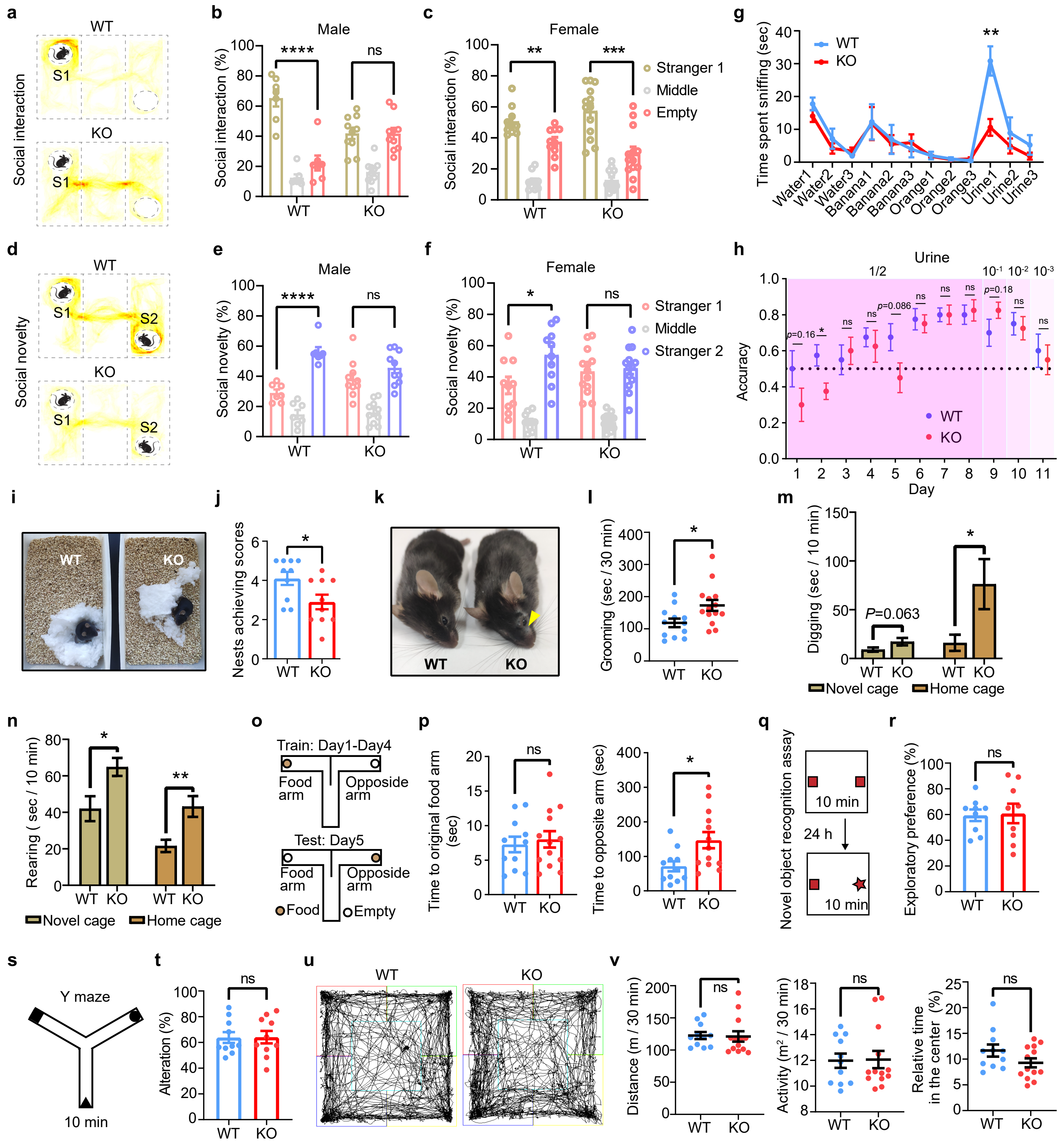

Autism-like behaviors in Sh3rf2 deletion mice. **a-c** Representative heatmap and quantitative results showing the relative time spent by WT and KO mice in each chamber during social interaction test phase in three chamber assay. Male: WT: n = 7 mice; KO: n = 10 mice. Female: WT: n = 10 mice; KO: n = 13 mice. t test. **d-f** Representative heatmap and quantitative results showing the relative time spent by WT and KO mice in each chamber during social novelty test phase in three chamber assay. Male: WT: n = 7 mice; KO: n = 10 mice. Female: WT: n = 10 mice; KO: n = 13 mice. t test. **g** Time spent sniffing scents in social olfactory assay. WT: n = 8 mice; KO: n = 9 mice. Multiple t test. **h** The quantitative result of olfactory threshold test showing normal olfactory detection threshold but aversion to the conspecific urine scent in KO mice. n = 8 mice per group. Mann-Whitney test. **i**, **j** Impaired nesting behavior in KO mice. n = 10 mice for all groups. Mann-Whitney U test. **k** Fur damage around whisker-area of KO mice. **l** Time spent grooming in open field within 30 min. n ≥ 12 mice for all groups. t test. **m**, **n** Time spent digging and rearing in home cage and novel cage within 10 min. n ≥ 10 mice for all groups. Mann-Whitney U test. **o**, **p** The schematic diagram of T maze assay and the time spent reaching the original “food arm” (left) and the “opposite arm” (right) in T maze assay. n ≥ 11 mice for all groups. t test. **q**, **r** Similar exploratory preference of WT and KO mice in novel object recognition assay. n = 9 mice for all groups. t test. **s**, **t** Similar alternation index of WT and KO mice in Y maze assay. n = 10 mice for all groups. t test. **u** Representative trajectories of WT and KO mice in open field within 30 min. **v** Comparable total moving distance, activity and time spent in the central area of WT and KO mice in open field within 30 min. n ≥ 11 mice for all groups. t test. All data are presented as mean ± SEM; *p < 0.05，**p< 0.01, ***p < 0.001, ****p < 0.0001; ns: no significance.
