# Supplementary material for: Autism-related proteins form a complex to maintain the striatal asymmetry in mice
Source: Cell Res. 2025 Sep 2;35(10):762–74. doi: 10.1038/s41422-025-01174-9 (PMC12485048; doi:10.1038/s41422-025-01174-9)
Supplement: Supplementary file 8 — Supplementary information, Figure S8 [file 41422_2025_1174_MOESM8_ESM.pdf]

Supplementary Figure 8

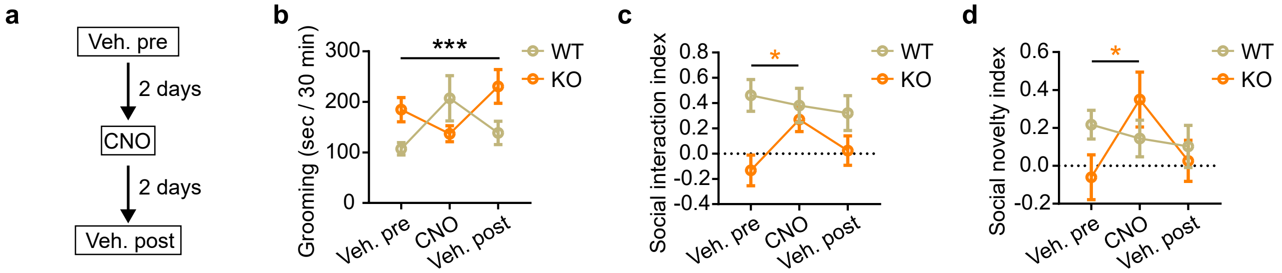

Rescue autism-like behaviors in Sh3rf2 deletion mice with DREADD. **a** Flow chart of the alternating paradigm of behavior test. The WT and KO mice here were the same as those treated with Veh in Figure 6B. n = 8-10 mice for each group. **b** Quantitative results of time spent grooming by WT and KO mice treated with Veh or CNO in open field within 30 min. Alternating paradigm. Two-way ANOVA. The interaction effect of genotype with treatment was significant. **c**, **d** Quantitative results of social interaction index (**c**) and social novelty index (**d**) of WT and KO mice treated with Veh or CNO. Three chamber assay with alternating paradigm. Two-way ANOVA with Dunnett's multiple comparisons test. The differences of social interaction index and social novelty index between KO-Veh.pre group and KO-CNO group were significant. All data are presented as mean ± SEM; *p < 0.05; ***p < 0.001; ns: no significance.
