# Supplementary material for: Autism-related proteins form a complex to maintain the striatal asymmetry in mice
Source: Cell Res. 2025 Sep 2;35(10):762–74. doi: 10.1038/s41422-025-01174-9 (PMC12485048; doi:10.1038/s41422-025-01174-9)
Supplement: Supplementary file 13 — Supplementary information, Table S5 [file 41422_2025_1174_MOESM13_ESM.pdf]

Table S5. Antibodies used in this paper

Antibodies	Source	Identifier
Chicken anti-LacZ	Abcam	Cat#ab9361; RRID: AB_307210
Mouse anti-NeuN	Abcam	Cat#ab104224; RRID: AB_10711040
Mouse anti-Calbindin-D-28K	Sigma	Cat#C9848; RRID: AB_476894
Rabbit anti-mCherry	Abcam	Cat#ab167453; RRID: AB_2571870
Mouse anti-HA	MBL	Cat#M180-3; RRID: AB_10951811
Mouse anti-Flag	MBL	Cat#M185-3L; RRID: AB_11123930
Chicken anti-GFP	Abcam	Cat#ab13970; RRID: AB_300798
Rabbit anti-ChAT	Abcam	Cat#ab178850; RRID: AB_2721842
Mouse anti-PV	Sigma	Cat#P3088; RRID: AB_477329
Rabbit anti-SOM	IMMUNOSTAR	Cat#20067; RRID: AB_572264
Rabbit anti-DARPP32	Abcam	Cat#ab40801; RRID: AB_731843
Rabbit anti-α-tubulin	Abclonal	Cat#ac007; RRID: AB_2772755
Mouse anti-α-tubulin	CST	Cat#3873s; RRID: AB_1904178
Rabbit anti-CaMKII	Abcam	Cat#ab134041; RRID: AB_2811181
Rabbit anti-P-CaMKII(Thr286/Thr287)	Abcam	Cat#ab124880; RRID: AB_10974848
Rabbit anti-PPP1CC	Abclonal	Cat#A4035; RRID: AB_2863175
Rabbit anti-PPP1CA+1CB	Abcam	Cat#ab52619; RRID: AB_2170391
Mouse anti-GluR1	Millipore	Cat#MAB2263; RRID: AB_11212678
Rabbit anti-P-GluR1(Ser831)	Millipore	Cat#04-823; RRID: AB_1977218
Rabbit anti-GluR2	Abcam	Cat#ab133477; RRID: AB_2620181
Rabbit anti-NR1	CST	Cat#5704s; RRID: AB_1904067
Rabbit anti-NR2A	CST	Cat#4205s; RRID: AB_2112295
Rabbit anti-NR2B	CST	Cat#4212s; RRID: AB_2112463
Rabbit anti-PSD-95	CST	Cat#3450s; RRID: AB_2292883
Rabbit anti-SYN	Abcam	Cat#ab14692; RRID: AB_301417
Mouse anti-β-actin	CST	Cat#3700s; RRID: AB_2242334
