# Supplementary material for: Autism-related proteins form a complex to maintain the striatal asymmetry in mice
Source: Cell Res. 2025 Sep 2;35(10):762–74. doi: 10.1038/s41422-025-01174-9 (PMC12485048; doi:10.1038/s41422-025-01174-9)
Supplement: Supplementary file 14 — Supplementary information, Data S1 [file 41422_2025_1174_MOESM14_ESM.pdf]

Supplementary information, Data S1

Materials and Methods

Three-chamber test

The three-chamber apparatus consists of three equally sized chambers with two small doors. Each chamber can be closed and opened with a door. Wild-type mice of the same sex and age were used as Stranger 1 and 2. In habituation phase, a wire cage was placed in the left and right chambers respectively, and the subjected mouse was put in the middle chamber and allowed to explore three chambers freely for 5 min. In social interaction test phase, Stranger 1 was introduced into the wire cage in the left or right chamber randomly. Then, two entrances were opened to allow the subjected mouse to explore freely for 5 min. Following 5 min, in the social novelty phase, Stranger 2 was introduced into the previously empty wire cage, and two entrances were again opened to allow the subjected mouse to explore freely for 5 min. Time spent in each chamber and the subjected mouse's trajectory was recorded using SMART Video Tracking System (Panlab). The social interaction index was calculated by subtracting the time spent interacting with Stranger 1 from the time spent in contact with the empty wire cage and dividing by their sum. The social novelty index was calculated by subtracting the time spent interacting with Stranger 2 from the time spent interacting with Stranger 1 and dividing by their sum.

Free dyadic social interaction

The subjected mouse was housed individually 3 days before the testing day. An unfamiliar wild-type mouse of the same sex, age and weight was introduced. The two mice were allowed to interact freely and videotaped continuously for 10 min.

Olfactory function test

The subjected mouse was exposed to each scent in three consecutive 3-min trials in the following order: water, banana (banana milk drink, Sanyuan), orange (Orange juice, Fanta), and urine (collected from at least 7 age- and gender-matched unfamiliar mice, diluted 1:1 with water). Time spent sniffing was recorded by an examiner with a stopwatch.

Olfactory Sensitivity Test Protocol

We employed an operant conditioning paradigm within a modified three-chamber apparatus to assess olfactory detection thresholds for unfamiliar, age- and gender-matched mouse urine. The custom apparatus featured two end chambers equipped with automated one-way access doors, liquid sample presentation dishes, and precise food pellet dispensers. An automated system restricted door reopening following chamber entry. One day prior to testing, mice received a ration of chow (1.5 g per 20 g body weight). The protocol comprised three sequential phases: Habituation (2 days): Mice underwent 5 daily trials (10 trials total) where both end-chamber dishes contained 2× saline-diluted urine. Entry into either chamber triggered chow reward. Training: Mice received daily sessions of 5 trials. Randomly assigned end-chambers contained either 2× saline-diluted urine (S+) or saline alone (S-). Entry into the S+ chamber yielded a reward; entry into S- resulted in immediate removal without reward. Trials not initiated within 3 min were recorded as incorrect. Training continued until each mouse achieved ⩾80% correct responses one day. Threshold Testing: Mice underwent 5 daily trials as described for training, but with ascending serial dilution of the urine stimulus (10×, 100×, 1000×) presented sequentially across days. Testing concluded upon a subject's performance dropping to approximately 50% correct for a given dilution, indicating the olfactory detection threshold.

Nest building assay

Briefly, the subjected mouse was housed individually and given one piece of cotton fiber (5 cm × 5 cm) as a nest building material. The quality of the nest was scored 16 h later.

Open field

Open field test was conducted in a square box (50 cm × 50 cm) for 30 min. Trajectory and time spent in the center quadrant (25 cm × 25 cm) were recorded using SMART Video Tracking System (version 3.0, Panlab). Meanwhile, self-grooming time was recorded by an examiner with a stopwatch.

Digging and Rearing in novel cage and home cage

Time spent by the subjected mouse in digging and rearing was recorded in a novel cage filled with a thin-layer of clean bedding for 10 min. The mouse was then returned to the home cage filled with bedding, and its digging and rearing behaviors were recorded for 10 min immediately.

T maze test of stereotyped behavior

It was performed as previously described with minor modifications (*63*). Briefly, it consists of training phase (4 days) and test phase (1 day). Before the experiment, the left or right arm of the T maze was defined as the "food arm" randomly for each mouse (once determined, it could not be changed during the whole experiment). On the evening of the previous day, each mouse mice received a ration of chow (1.5 g / 20 g). The amount of food could be appropriately added or reduced according to the changes in the body weight of the mice under the precondition that the body weight of the mice was not less than 85% of the initial body weight during the whole experiment. During the training phase, a small chow piece was placed at the end of the "food arm", and the mouse was placed on the vertical arm and allowed to explore freely. If the mouse entered the "food arm" and touched the food, it was considered that the choice was correct, and the mouse was put back into the cage and rewarded to eat for 3 min. If the mouse entered and reached the end of the contralateral arm ("opposite arm"), or if the mouse did not make a choice within 3 min, it was considered a failure and the mouse was returned to the cage without reward. Each mouse was trained 5 times a day. Then, the mouse was given enough time to eat enough food. When the mice had finished eating, all the left food was taken away, and the mouse was given a ration of chow. In the test phase on day 5, a chow piece was placed on the "opposite arm". The mouse was placed on the vertical arm and allowed to explore freely. The time spent reaching the end of the original "food arm" and the "opposite arm" was recorded.

Y maze test

It is used to assess the short-term memory in mice. The subjected mouse was placed in the middle of the Y maze marked with different shapes (circle, square, triangle) at the ends of the three arms, and the order in which the mouse entered the three arms was recorded for 10 min. If the mouse entered three different arms in succession, this was defined as one alternation. The alternation index was calculated as total alternations divided by (total entries - 1). Higher alternation index indicates better short-term memory.

Novel object recognition assay

It was performed in the open field box. The subjected mouse was familiarized with two identical objects placed in the box (one left and one right) for 10 min, and was tested for the long-term memory 24 h later. One of objects was replaced by a new different object. The exposure time of the mouse to the old and new objects was recorded for 10 min. The exposure was defined as direct contact with the object with the nose of mouse or as that the mouse was oriented toward the object and standing within 2 cm of the object. The exploratory preference was calculated as the exposure time of the new object divided by total exposure time.
